# Altered Sphingolipid Metabolism is Associated with Osimertinib Resistance in Nonsmall-Cell Lung Cancer

**DOI:** 10.1021/acs.jproteome.6c00216

**Published:** 2026-04-16

**Authors:** Julia Babuta, Aleksandra Gruevska, Chiharu Wickremesinghe, Alex Montoya, Georgia Roumelioti, Pavel Shliaha, Cristina Balcells, Flora McKinney, Chandler Bray, Toby Athersuch, Matthew Martin, Hector Keun, Zoe Hall

**Affiliations:** † Department of Metabolism, Digestion and Reproduction, 4615 Imperial College London, London W12 0NN, United Kingdom; ‡ Department of Surgery and Cancer, Imperial College London, London W12 0NN, United Kingdom; § MRC Laboratory of Medical Sciences, London W12 0NN, United Kingdom; ∥ 468087 AstraZeneca, Biomedical Campus, Cambridge CB2 0AA, United Kingdom

**Keywords:** nonsmall-cell lung cancer, lipidomics, glycosphingolipid, ceramide, sphingolipid, drug resistance, multiomics, glucosylceramidase

## Abstract

Nonsmall-cell lung cancer (NSCLC) accounts for more than 80% of lung cancer cases. Epidermal growth factor receptor mutations (EGFRm) occur in 15 and 40% of NSCLC in Western and Asian populations, respectively. Current treatment for advanced NSCLC targets EGFRm with tyrosine kinase inhibitors (TKIs). Osimertinib is a third-generation EGFR-TKI now used as a first-line treatment in advanced/metastatic NSCLC; however, drug resistance frequently develops. Dysregulation of metabolism has been suggested to play a role in the development of drug resistance. Here, we investigated the role of lipid metabolism in the development of osimertinib resistance (OR) using pharmacologically-induced resistant cellular models. We used a multiomics approach, combining lipidomics with proteomics analyses. We found alterations in processes relating to metabolism, such as dysregulated sphingolipid metabolism. In particular, we identified that OR lines reduce free ceramides in favor of complex glycosphingolipids. Mechanistically, this metabolic shift avoids ceramide-mediated apoptosis via caspase-3 activation. Importantly, when we combined osimertinib with D-PDMP, an inhibitor of the key enzyme responsible for the conversion of ceramide to glucosylceramide, we increased the sensitivity to osimertinib. Overall, we have identified the glycosphingolipid metabolic pathway as a potential therapeutic target to reinstate sensitivity to osimertinib in NSCLC.

## Graphical abstract

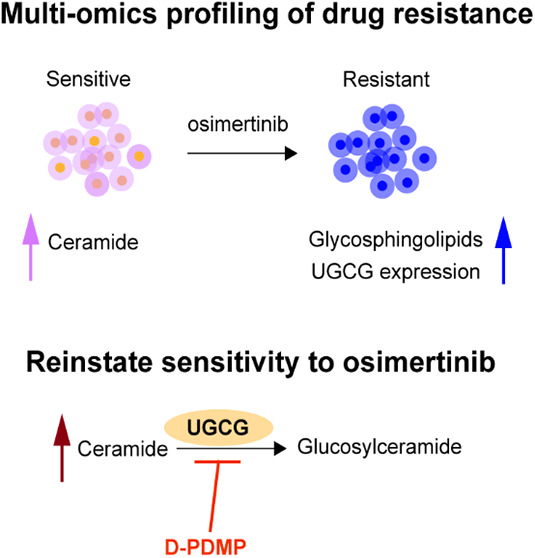

## Introduction

Lung cancer is the primary cause of cancer-related deaths globally.^1^ Nonsmall-cell lung cancer (NSCLC) comprises around 85% of all lung cancer cases, with a 5-year survival rate of 27%.^2^ Epidermal growth factor receptor mutations (EGFRm) are the most common mutations in NSCLC^3^ and are druggable targets. EGFR-tyrosine kinase inhibitors (EGFR-TKIs) have been developed to target common mutations within EGFR. First-generation EGFR-TKIs include gefitinib and erlotinib; however, patients typically acquire resistance rapidly by developing a T790M point mutation within the EGFR. To counter this and further mutations, second- and third-generation EGFR-TKIs have been developed. Osimertinib is a third-generation EGFR-TKI;^4^ however, most patients will eventually develop resistance.

Drug resistance is multifaceted and often results from a combination of genetic and nongenetic factors. Nongenetic factors include increased drug efflux, altered drug metabolism, and modulation of the tumor microenvironment. Altered metabolism is a hallmark of cancer and can also contribute to driving drug resistance. For instance, glutathione and amino acid metabolism were dysregulated in EGFR-TKI-resistant NSCLC.^5^ In addition, studies have shown that EGRF-TKI-resistant NSCLCs favor oxidative phosphorylation over glycolysis for energy production.^6^ Aberrant lipid metabolism has also been linked to EGFR-TKI resistance through an increase in de novo lipogenesis in resistant cells compared to sensitive ones.^7^ However, most studies have mainly been performed on previous generations of EGFR-TKIs, with limited data on the mechanisms behind osimertinib resistance.

Recently, sphingolipid metabolism was implicated in osimertinib resistance.^8^ Sphingolipids are a complex family of bioactive lipids, including ceramides, sphingomyelin, and complex glycosphingolipids.^9^ They are vital structural components within cell membranes and play a signaling role in physiological processes such as apoptosis and cell growth.^10^ Ceramides are central to sphingolipid metabolism; their accumulation activates caspase proteins that promote apoptosis, a highly regulated mechanism of programmed cell death.^11,12^ Ceramides are converted to glucosylceramide (GlcCer) by uridine-5-diphosphate (UDP)-glucose ceramide glucosyltransferase (UGCG) enzyme, also known as glucosylceramidase.^11^ UGCG overexpression has been documented in different cancers^13^ and is linked to various cellular processes, including multidrug resistance,^14^ increased oxidative phosphorylation, and mitochondrial turnover.^13^

Here, we investigated changes in lipid metabolism in pharmacologically induced models of osimertinib resistance (OR). Our data revealed that lipid metabolism was altered in OR cells compared to that in osimertinib-sensitive (OS) cells. In particular, OR cells showed decreased free ceramide with increased glycosphingolipid abundance. Inhibition of UGCG in OR cell lines, with D-PDMP, increased ceramide levels and triggered caspase-3-mediated apoptosis, acting synergistically with osimertinib. Targeting specific metabolic pathways can therefore be an approach to reinstate sensitivity to osimertinib in drug-resistant cells to allow continued benefit from osimertinib therapy.

## Materials and Methods

### Cell Culture and Dose–Response Assays

All cell lines were kindly provided by the Innovative Medicines team at AstraZeneca (Cambridge, U.K.). Pharmacologically induced resistant cell lines were generated by chronically dosing the PC9 parental cell line (PC9P) with increasing concentrations of osimertinib to a maximum dose of 160 nM, producing two osimertinib-resistant (OR) clones, PC9R5 and PC9R6, previously described.^15^ PC9P was used as the osimertinib-sensitive (OS) control. All cell lines were grown in RPMI 1640 (Sigma), supplemented with 10% fetal bovine serum (ThermoFisher, 10270106), 2 mM l-glutamine (ThermoFisher, 25030-081), and 100 units/mL penicillin–streptomycin (ThermoFisher, 15070-063). The resistant lines (PC9R5 and PC9R6) grew under the same conditions as the parental lines upon the addition of 160 nM osimertinib (AZD9291; AstraZeneca). Cell lines were incubated at 37 °C with 5% CO_2_ and passaged when they reached 80% confluency. Cell lines were authenticated using Eurofins, and regular mycoplasma testing was performed.

Sulphorodamine B (SRB) assay was used to determine cell viability and inhibition of cell growth (details in Supporting Information Methods) by treating cells with varying concentrations of osimertinib (0.0001, 0.001, 0.01, 0.1, 1, and 10 μM), D-PDMP (5–50 μM; Cambridge Bioscience), C2-ceramide (*N*-Acetyl-d-sphingosine; 10–50 μM; Sigma-Aldrich), or vehicle control (0.1% DMSO, Sigma-Aldrich) for 72 h. Values were normalized to vehicle control.

### Combination Treatment of D-PDMP with Osimertinib

Each cell line was treated with doses of D-PDMP or C2-ceramide, with and without osimertinib, for 72 h according to its individually calculated IC_50_ value (Table S1). Cell growth was assessed using the SRB assay. Apoptosis was measured using the caspase-Glo 3/7 Assay following manufacturer guidelines. Luminescence was measured using a CLARIOstar Plate Reader (BMG Labtech). Caspase activity was background corrected using cell-free wells and normalized to the SRB cell mass data and vehicle control.

### Sample Preparation of Protein Pellets for Proteomic Analysis

Protein pellets following lipid extraction (see Supporting Information Methods) were resuspended through the addition of 150 μL of SEPOD surfactant cocktail buffer to the pellet and sonicating at 100% energy for 2 min on a Horn Sonicator before shaking at 1300 rpm on an orbital shaker at 70 °C, as previously described.^16^ The Pierce BCA Protein Assay Kit (ThermoFisher, 23225) assay^17^ was used to determine protein concentration and adjusted to 2g/L. One hundred fifty microliters of each sample (300 μg) were reduced and alkylated via the addition of 20 nM Tris­(2-carboxyethyl)­phosphine (TCEP) and 40 mM chloroacetamide. The samples were then subjected to precipitated-assisted capture (PAC) (16) via the addition of 1.5 mg of hydroxyl beads (MR-HYX010) and 450 μL of ethanol. The beads were washed with 500 μL of 80% ethanol and digested overnight in 300 μL of 50 mM ammonium bicarbonate buffer containing 20 ng/μL Trypsin and 10 ng/μL Lys-C while shaking at 1300 rpm in Eppendorf deep well plates. The resulting digest was then transferred to a new plate, and the resin was washed with 50 μL of 1% trifluoroacetic acid (TFA).

### Proteomics Analysis

The protein digests were analyzed by liquid chromatography mass spectrometry (LC-MS) using an UltiMate 3000 RSLC nanoliquid chromatography system coupled to an Exploris 240 MS (Thermo Scientific) via an EASY-Spray source. Electrospray nebulization was achieved by interfacing to Bruker PepSep emitters (PN: PSFSELF20, 20 μm). Peptides were separated using a 66 min stepped gradient method in the positive mode only using Acquity CSH C18 1.7 μM beads, 300 μM × 35 cm column, where 2 μg of digests resuspended in 5 μL was injected directly into the column at a flow rate of 5 μL/min for 4 min.^18^ Mobile phases were prepared with UPLC-grade solvents and chemicals (see Supporting Information Methods for further details). The peptides were then separated using a 66 min stepped gradient from 0–45% Mobile Phase B. The data-independent acquisition (DIA) mode was as follows: an initial MS1 scan with a mass range of 410–1650 *m*/*z* at 120,000 resolution with an AGC target of 3 × 10^6^ ions for a maximum injection time (IT) of 200 ms. This was followed by an MSX (boxcar) MS1 scan with 10 variable windows covering a range of 410–1650 *m*/*z* at 120,000 resolution and 310 DIA scans with variable window width at 30,000 resolution. The ACG target was again set to 3 × 10^6^ ions, with maximum IT set to auto. The normalized collision energy was set to 27%, and the total run acquisition time was 82 min.

### Proteomics Data Processing

Raw data were processed using Spectronaut v18.6.231227.55695 (Biognosys). Analysis was carried out using direct DIA. Library generation and database search were carried out with default settings for a trypsin/p specific digest: “Missed Cleavage Rate” was set to 3, and “Variable Modifications” allowed for methionine oxidation, protein N-terminal acetylation, asparagine deamidation, and cyclization of glutamine to glutamate. Peptide-spectrum matching (PSM) and protein group FDR were set to 0.01. Searches were carried out against UniProt *Homo sapiens* proteome (UP000005640) 1 gene per protein sequence database,^19^ which was accessed on 22/01/2024 and yielded 20,596 entries. Searches were also carried out against a universal protein contaminants database,^20^ which was accessed on 22/01/2024 and yielded 381 entries. For direct DIA analysis, a mutated decoy database was employed using a protein q-value cutoff set to 0.01 at the identification level. The quantification parameters were set to MS2 with the “Proteotypicity” filter set to “Only Proteotypic”. There was no value imputation strategy employed. The protein quantification method was set to MaxLFQ.^21^ The raw and local normalized protein intensity data generated were exported for further downstream analysis.

For downstream analysis, protein tables were filtered to remove the contaminant proteins. Protein accession IDs were matched to gene names using the UniProt ID mapping service.^22^ The Perseus platform v1.6.15.0 ^23^ was used for all further analysis. The data were log2-transformed prior to additional filtering and statistical analysis. Where multigroup comparisons were performed, data were filtered for proteins that contained ≥5 replicate intensities per experimental group (6 total replicates in each group). One-way ANOVA of the protein intensities was performed with multiple testing correction and permutation-based FDR set to 0.05, and these were then mean-centered (z-score normalized). Tukey’s HSD posthoc testing was carried out on ANOVA significant hits to determine pairs of groups, which were defined as having significant differences in means. For two-way group comparisons, the intensities were filtered as described, and Student’s *t*-test analysis was performed with multiple testing corrections and permutation-based FDR set to 0.05. The results were visualized as volcano plots.

### DAVID for Pathway Analysis of Proteomics Data

Proteomic data output from Perseus was analyzed via the Database for Annotation, Visualization, and Integrated Discovery (DAVID).^24,25^ Functional annotation and enrichment analysis were performed on proteins that were both statistically significant using ANOVA and Tukey’s posthoc testing in multiple comparisons and using Student’s *t*-test in two-way comparisons. Kyoto Encyclopedia of Genes and Genomes (KEGG) databases were selected for enrichment analysis. Pathways with FDR < 0.1 were considered significantly enriched.

### Analysis of Published Transcriptomics Data

Bulk transcriptomics data from Gene Expression Omnibus (GEO) were analyzed (GSE202859; https://www.ncbi.nlm.nih.gov/geo/). Resistance to osimertinib in HCC827, H1975, and PC9 cell lines was induced by growing the cells in increasing concentrations of drugs.^26^ Differential gene expression between each resistant cell line and its sensitive control was calculated using DESeq2 with the Benjamini–Hochberg correction for multiple testing.

### Lipid Extraction

Cells were cultured in 6 cm Cornell plates (six technical replicates) for 48 h. The media were removed, and the cells were washed with 1 mL of cold PBS. Ice-cooled methanol was added, and the cells were scraped and collected. The samples were then dried under a nitrogen flow. Dual-phase liquid–liquid extraction was used to separate polar and nonpolar fractions as described in the Supporting Information Methods.

### Lipidomics Experiments

Lipid extracts were analyzed by LC-MS using an UltiMate 3000 HPLC system coupled to an LTQ-Orbitrap Elite MS system (Thermo Scientific). A 10 min reverse-phase liquid chromatographic method was used to separate lipid species prior to detection by MS in both positive and negative ionization modes. Separation was achieved using an Acquity UPLC C18 BEH column, 130 Å, 1.7 μm, 2.1 mm × 50 mm, 1/pk (Waters Corporation). Mobile phases were prepared with UPLC-grade solvents and chemicals as follows: A = 60% ACN, 40% H_2_O with 10 mM ammonium formate. B = 90% IPA, 10% ACN with 10 mM ammonium formate. C = 60% ACN, 40% H_2_O, and 10 mM ammonium acetate. D = 90% IPA, 10% ACN, and 10 mM ammonium acetate. The mobile phase gradient elution for positive and negative 10 min reverse-phase modes can be found in the Supporting Information Methods (Table S2). Five microliters of each sample was injected in the positive mode, and 10 μL was injected in the negative mode, both at a flow rate of 0.5 mL/min at a column temperature of 55 °C. The spectra were acquired in both ionization modes in the range of 110–2000 *m*/*z* at 60,000 mass resolution.

Data were acquired using Xcalibur (Thermo) and converted from a raw format to mzML via ProteoWizard MS Convert software.^27^ Peak picking was performed using the XCMS package in R studio (Version R4.2.2.2). Data were normalized to the total ion count, and univariate scaling was performed. Lipids were annotated based on retention time and accurate mass database searches in Lipid Maps “Bulk” Structure Searches Database (LMSD).^28^ Metaboanalyst v6.0 ^29^ was used for hierarchical clustering with the Euclidean algorithm for distance measure, the Ward algorithm for clustering measure, and ANOVA for testing significance.

### Synergy Analysis of Combination Therapy

Synergy data analysis was performed on the drug combination data of D-PDMP and osimertinib using the web application SynergyFinder+^30^ with the Bliss Independence model of combination analysis. The cutoffs for Bliss scoring we used were as follows: less than −5 for antagonistic, −5 to 5 for additive, and >5 for synergistic.^31^ Further details are found in Supporting Information Methods.

## Results

### Osimertinib Resistance in Cellular Models

PC9 parental (PCP9) cell lines were treated with increasing doses of osimertinib, to a final dose of 160 nM, resulting in the generation of two pharmacologically induced resistant clones, PC9R5 and PC9R6. Both OR cell lines contain a deletion within exon 19 at E746-A750, as present in the PC9 parental cell line. PC9R5 additionally had gain-of-function mutations in MAPK1 and CRKL (druggable targets activating the RAS-RAF-MAPK pathway), as previously described.^15^

To confirm and characterize the models’ acquired resistance to osimertinib, OR and OS cell lines were treated with increasing doses of osimertinib and growth was measured (Figure S1). As expected, OR cell lines were more resistant to osimertinib, with larger calculated IC_50_ values (1.7 and 1.9 μM for PC9R5 and PC9R6, respectively) than the PCP9-sensitive control (0.003 μM).

### Proteomics Reveals Altered Metabolic Processes in Osimertinib Resistance

To determine potential altered biological pathways in osimertinib resistance, we carried out untargeted proteomics on the two OR cell lines and a PC9P-sensitive control. We detected over 7,000 proteins from which 6,186 were identified by 2 or more peptides (Table S3). Overall, there were 5,786 proteins that were statistically different across the three groups. The top 50 KEGG pathways that were enriched within the significantly changed protein set included metabolism-centric pathways such as the TCA cycle, amino sugar and nucleotide metabolism, fatty acid degradation, and fatty acid metabolism (Figure 1A).

**Figure 1:**
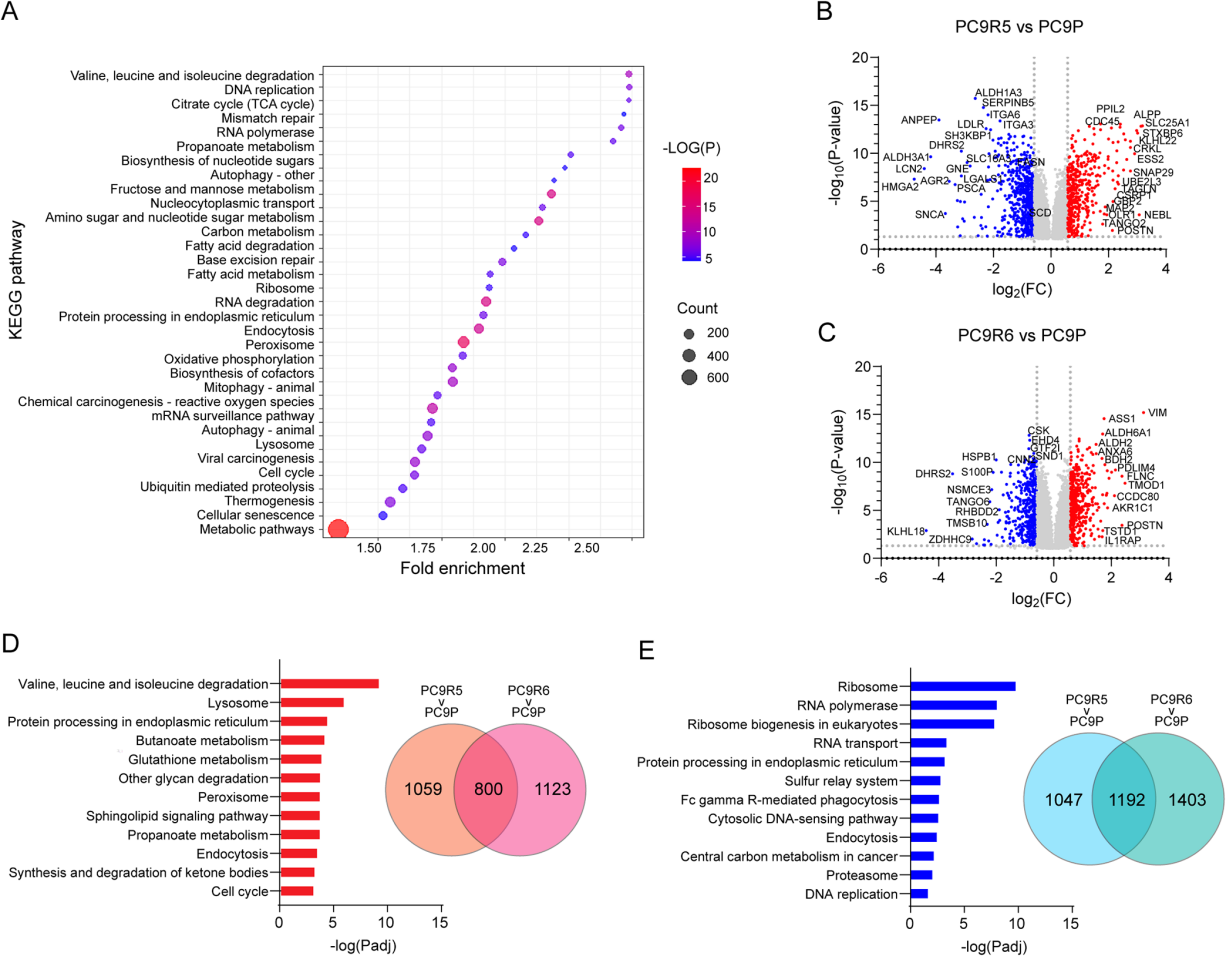
Untargeted proteomic analysis of pharmacologically induced models of osimertinib resistance (PC9R5 and PC9R6) and sensitive control (PC9P). KEGG pathways enriched in proteins differentially expressed across the three cell lines (A). Two-way comparisons of protein changes in (B) PC9R5 compared to PC9P and (C) PC9R6 compared to PC9P. Volcano plot showing proteins that are significantly (fold change >1.5 and FDR-adjusted *p*-value <0.05) increased (red) and decreased (blue). Data were normalized via local regression normalization. Overlap of upregulated (D) and downregulated (E) proteins in each OR cell line (PC9R5 and PC9R6) versus PC9P-sensitive control, and the top 12 enriched KEGG pathways for the common altered proteins. Technical replicates, *n* = 6; data shown for one of two biological replicate experiments.

We next performed two-way comparisons of each OR cell line compared to the sensitive control. Between PC9R5 (OR) and PC9P (OS), there were 4,099 proteins significantly different based on Student’s *t*-test and Tukey’s HSD posthoc testing for multiple comparisons (Figure 1B). Downregulated proteins in OR included members of the aldehyde dehydrogenase families (ALDH1A3 and ALDH3A1), which play key roles in lipid metabolism and peroxidation,^32^ SH3 domain containing kinase binding protein 1 (SH3KBP1), which is involved in apoptosis,^33^ low-density lipoprotein receptor (LDLR), which regulates cholesterol levels,^34^ and dehydrogenase/reductase 2 (DHRS2), which is involved in lipid metabolism and redox regulation.^35^ Upregulated proteins included solute carrier 25A1 (SLC25A1), which is a mitochondrial carrier and is involved in the movement of citrate across membranes,^36^ and CRK-like proto-oncogene (CRKL), which activates the RAS/RAF/MAPK signaling pathway.^15,37^ The full list of significantly changed proteins is found in Table S4.

We then compared the proteome of PC9R6 (OR) to PC9P (OS). Four thousand five hundred nineteen proteins in this comparison were significantly different (Figure 1C and Table S5). Major downregulated proteins included HPSB1, which has a protective effect against apoptosis,^38^ and DHRS1. Major upregulated proteins included ALDH6A1 and ALDH2, which function to protect cells from lipid peroxidation-derived aldehydes. Other upregulated proteins included ASS1, which catalyzes the penultimate step in arginine biosynthesis,^39^ BDH2, which is a short-chain dehydrogenase/reductase and regulates intracellular reactive oxygen species and iron levels,^40^ and glutathione peroxidase 3 (GPX3), which protects cells from oxidative stress.^41^

With more than 4000 significantly altered proteins, it is critical to assess pathway coherence and effect size to aid biological interpretability. Therefore, to identify common pathways altered in OR, we first determined the overlap of significantly altered proteins in each OR cell line compared to the sensitive control. We identified 800 common upregulated proteins with 1192 shared downregulated proteins (Figure 1D,E). Next, we performed enrichment analysis to determine the shared altered pathways (KEGG pathway terms). The top 10 enriched pathways for upregulated proteins included valine, leucine, and isoleucine degradation (fold enrichment = 6.9, *p*_adj_ = 5.7 × 10^–10^), glutathione metabolism (fold enrichment = 4.0, *p*_adj_ = 1.2 × 10^–4^), sphingolipid signaling pathway (fold enrichment = 2.73, *p*_adj_ = 1.8 × 10^–4^), and cell cycle (fold enrichment = 2.4, *p*_adj_ = 7.0 × 10^–4^) (Figure 1D). The top enriched pathways for downregulated proteins included ribosome (fold enrichment = 3.1, *p*_adj_ = 1.2 × 10^–10^) and central carbon metabolism in cancer (fold enrichment = 2.7, *p*_adj_ = 0.005) (Figure 1E).

### Lipid Metabolism is Dysregulated in Pharmacological Models of Osimertinib Resistance

Given the proteomic changes in fatty acid and lipid metabolism, we carried out untargeted lipidomics analysis to investigate the alteration of lipid metabolism in the resistant cell lines. Lipids were extracted and analyzed by LC-MS/MS. Overall, there were 1415 unique features in positive and negative ion modes that were retained after quality control; from these, 404 lipids were annotated. Hierarchical clustering of the lipid profiles distinguished the three cell lines, recognizing lipidomic differences between OR and OS and between the two resistant cell lines, PC9R5 and PC9R6. The top 50 features significantly different, based on ANOVA, across the 3 cell lines included plasmalogens, triacylglycerides (TAGs), and glycosphingolipids (Figure 2A).

**Figure 2:**
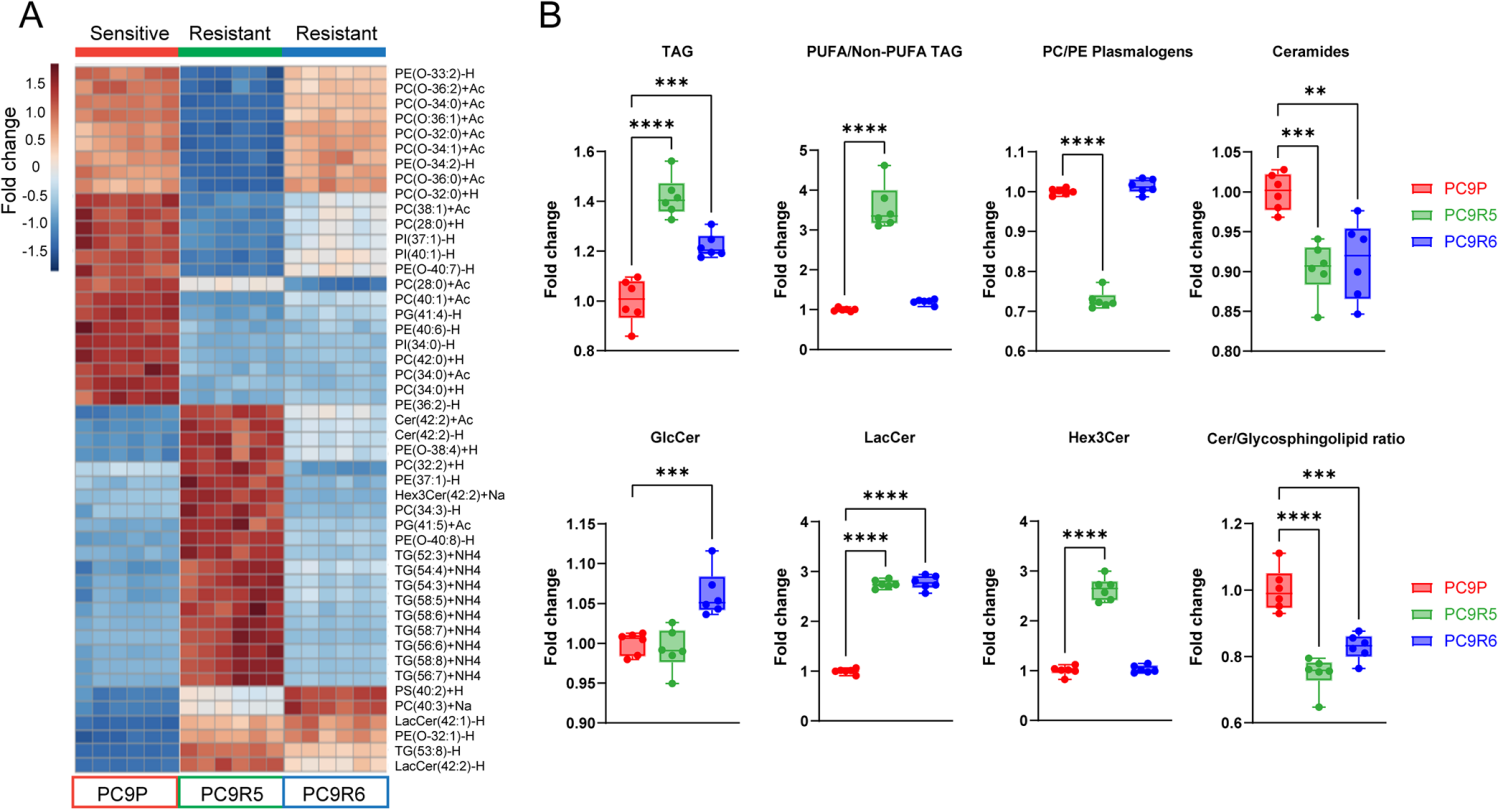
Untargeted lipidomics of cell models of osimertinib resistance (OR; PC9R5 and PC9R6) and sensitive control (OS; PC9P). (A) Hierarchical clustering of lipid profiles revealed differences between the 3 cell lines. The heatmap shows the top 50 features differentiating the 3 cell lines (based on ANOVA). (B) Lipid class fold change for OR cell lines relative to OS, including total TAGs, ratio of PUFA/Non-PUFA TAG, total PC and PE plasmalogen abundance, total ceramide, glucosylceramide (GlcCer), lactosylceramide (LacCer), trihexosylceramide (Hex3Cer), and the ratio of free ceramide to total glycosphingolipid abundance. Statistical differences in box plots were determined by one-way ANOVA with Dunnett’s correction for multiple comparisons. ns, *p* > 0.05; *p < 0.05; **p < 0.01; ***p = 0.001; ****p < 0.0001 compared with the control. Technical replicates, *n* = 6; data shown for one of two biological replicate experiments.

We plotted the total intensity of key families of lipids that were important in driving the differences between the groups (Figure 2B). The abundance of total TAG was increased in both OR cell lines compared to OS. We also found changes in the TAG composition, with increased polyunsaturated fatty acid (PUFA) containing TAG in PC9R5, compared to both PC9P and PC9R6. On the other hand, PC9R5 had a significant decrease in total plasmalogens compared to the OS control, whereas plasmalogens were unchanged in PC9R6 (Figure 2B). Finally, in both OR cell lines relative to the control, we discovered a decrease in total free ceramides and an increased abundance of complex glycosphingolipids, such as lactosylceramide (LacCer) (Figure 2B).

Next, we specifically explored the differential expression for proteins known to be involved in lipid metabolism according to the KEGG database (Table S6). The increased TAG species in OR points to an increase in lipid synthesis. However, key proteins related to fatty acid synthesis were downregulated in both OR cell lines, for example, FASN and SCD (Figure 3A). On the other hand, SCD5 was increased in both cell lines. ELOVL5, associated with fatty acid elongation, was decreased in PC9R5 only, while FADS1, linked to PUFA metabolism, was significantly decreased in both cell lines. Neither of these changes would account for the increased PUFA/Non-PUFA TAG in the PC9R5 cell line. However, consistent with the decrease in plasmalogen lipid abundance in PCPR5, decreased FAR1 and FAR2, rate-limiting proteins in plasmalogen synthesis,^42,43^ were observed in both OR cell lines (Figure 3A).

**Figure 3:**
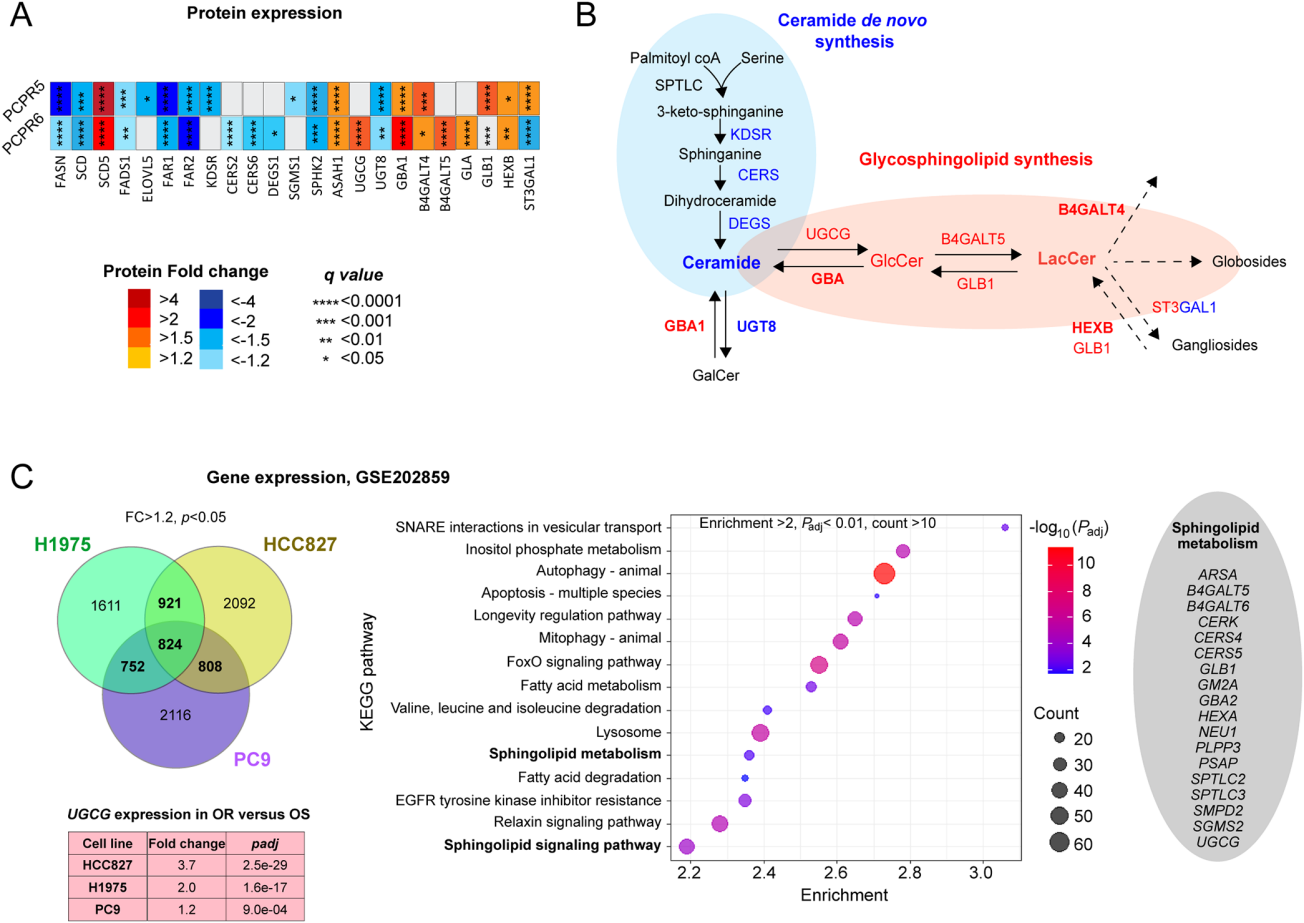
Lipid metabolism is altered at the protein level in osimertinib resistance. (A) Abundance fold change relative to PC9P control for PCPR5 and PCPR6 OR cell lines for proteins pertaining to fatty acid, plasmalogen, ceramide, and glycosphingolipid metabolic pathways. Significance is based on Student’s *t*-test with false discovery correction (*q*-value). (B) Ceramide and glycosphingolipid synthesis pathways, with significantly changed protein and lipid changes (OR versus OS) indicated in red (increased) and blue (decreased). Names in bold indicate that consistent changes were found in both OR cell lines. (C) Analysis of published transcriptomics data sets of OR in 3 different cell lines revealed alterations in sphingolipid metabolism and signaling, and a significant increase in *UGCG* expression across all 3 cell lines.

Proteins involved in sphingolipid metabolism also had altered expression (Figure 3A,B). Proteins with decreased expression in PC9R5 compared to PC9P-sensitive control included PLPP2 (dephosphorylates ceramide-1-phosphate), KDSR (involved in de novo synthesis of ceramide), SPHK2 (converts sphingosine into sphingosine kinase), and UGT8 (converts galactosylceramide to ceramide). GLB1 and ST3GAL1 (both involved in the ganglioside metabolic pathway) had significantly increased expression in PC9R5. In PC9R6 versus PC9P-sensitive control, downregulated proteins included KDSR, CERS2, CERS6, and DEGS1, all involved in de novo ceramide synthesis. Upregulated proteins included UGCG, which catalyzes the conversion of ceramide to glucosylceramide, the rate-determining step in the formation of downstream glycosphingolipids. B4GALT5, involved in LacCer synthesis, was also significantly increased in the level of PCPR6.

In addition, we analyzed publicly available transcriptomics data sets for two further cell lines, HCC827 and H1975, and PC9 (GSE202859; Figure 3C). These are all human-derived NSCLC cell lines frequently used for investigating EGFR-TKI resistance. Resistance to osimertinib in HCC827, H1975, and PC9 was induced by growing the cells in increasing concentrations of drugs.^26^ Genes with expression significantly increased in 2 out of 3 cell lines were used as input for pathway enrichment analysis (Figure 3C). The top enriched KEGG pathways included “Sphingolipid metabolism” (Fold enrichment = 2.4, *p*_adj_ = 5.8 × 10^–3^) and “Sphingolipid signaling pathway” (Fold enrichment *=* 2.2, *p*_adj_ = 7.5 × 10^–5^). Upregulated genes in the “Sphingolipid metabolism” gene set included *UGCG, B4GALT5*, *B4GALT6* (involved in LacCer synthesis), among others. Of note, *UCGC* had significantly increased expression in OR versus OS across all three cell lines (Figure 3C).

### Sphingolipid Metabolism as a Therapeutic Target to Reinstate Osimertinib Sensitivity

Proteomic and lipidomic analyses both revealed alterations in sphingolipid metabolism in the OR. A decrease in free ceramide was found in both OR cell lines compared with OS, and an increase in glycosphingolipids, such as LacCer. The accumulation of ceramide can activate apoptosis via caspase-3. Decreased ceramide could assist the OR cells to evade apoptosis and thereby contribute to drug resistance. To test this, we treated OR and OS control cell lines with UGCG inhibitor D-PDMP for 72 h. UCGC is selected for inhibition, since it catalyzes the rate-determining step in the formation of glycosphingolipids such as lactosylceramides, globosides, and gangliosides and is immediately downstream of ceramide, with a strong basis in the literature for its inhibition increasing ceramide-mediated apoptosis.^8^

First, we assessed the combination of D-PDMP and osimertinib and its effect on cell growth to determine if D-PDMP resensitized the OR cell lines to osimertinib. For each cell line, we treated with a range of osimertinib doses (0.001–10 μM) to encapsulate the therapeutic dose (160 nM) and higher doses. We treated with three doses of D-PDMP: the cell-specific IC_50_ concentration (Figure S2), one lower dose (LD), and one higher dose (HD). In the PC9P cell line, increasing doses of D-PDMP in the absence of osimertinib resulted in a significant reduction in growth (Figure 4A). With the increasing dose of osimertinib monotherapy, there was a dramatic decrease in cell growth, as expected. In addition, there was a further significant effect on cell growth with the addition of D-PDMP in the presence of osimertinib. Interestingly, for PC9R5 (Figure 4B) and PC9R6 cell lines (Figure 4C), co-treatment of D-PDMP with osimertinib significantly inhibited cell growth when compared to osimertinib alone at a range of osimertinib doses. Importantly, this effect was also observed at lower doses of osimertinib, where monotherapy had a negligible effect on cell growth. We used LC-MS/MS to measure ceramide and glycosphingolipid levels and confirmed, as expected, that total glycosphingolipids were significantly increased relative to free ceramide, with D-PDMP treatment in combination with osimertinib (Figures 5 and S3).

**Figure 4:**
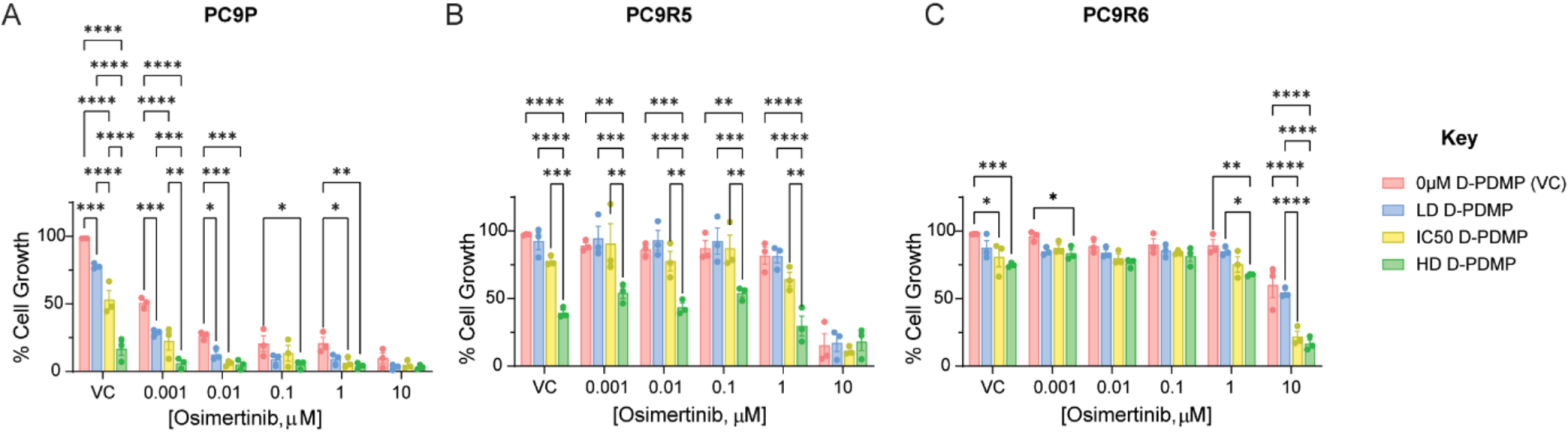
D-PDMP treatment in combination with osimertinib. Percentage (%) change in growth, measured via an SRB assay, after cells were treated with various combinations of D-PDMP and osimertinib for 72 h. (A) PC9P, (B) PC9R5, and (C) PC9R6 cells were treated with DMSO vehicle control (VC), osimertinib monotherapy, D-PDMP monotherapy, and combinations of both drugs for 72 h. D-PDMP LD/IC_50_/HD were 15/35/50 μM, 10/25/50 μM, and 5/15/30 μM for PC9P, PC9R5, and PC9R6 cell lines, respectively. Statistical differences in box plots were determined by two-way ANOVA with Dunnett’s correction for multiple comparisons. ns, *p* > 0.05; *p < 0.05; **p < 0.01; ***p = 0.001; ****p < 0.0001. For clarity, significance is only shown on the plot within each osimertinib concentration group. Data expressed as mean ± SEM. Technical replicates, *n* = 6; biological replicates, *n* = 3.

**Figure 5:**
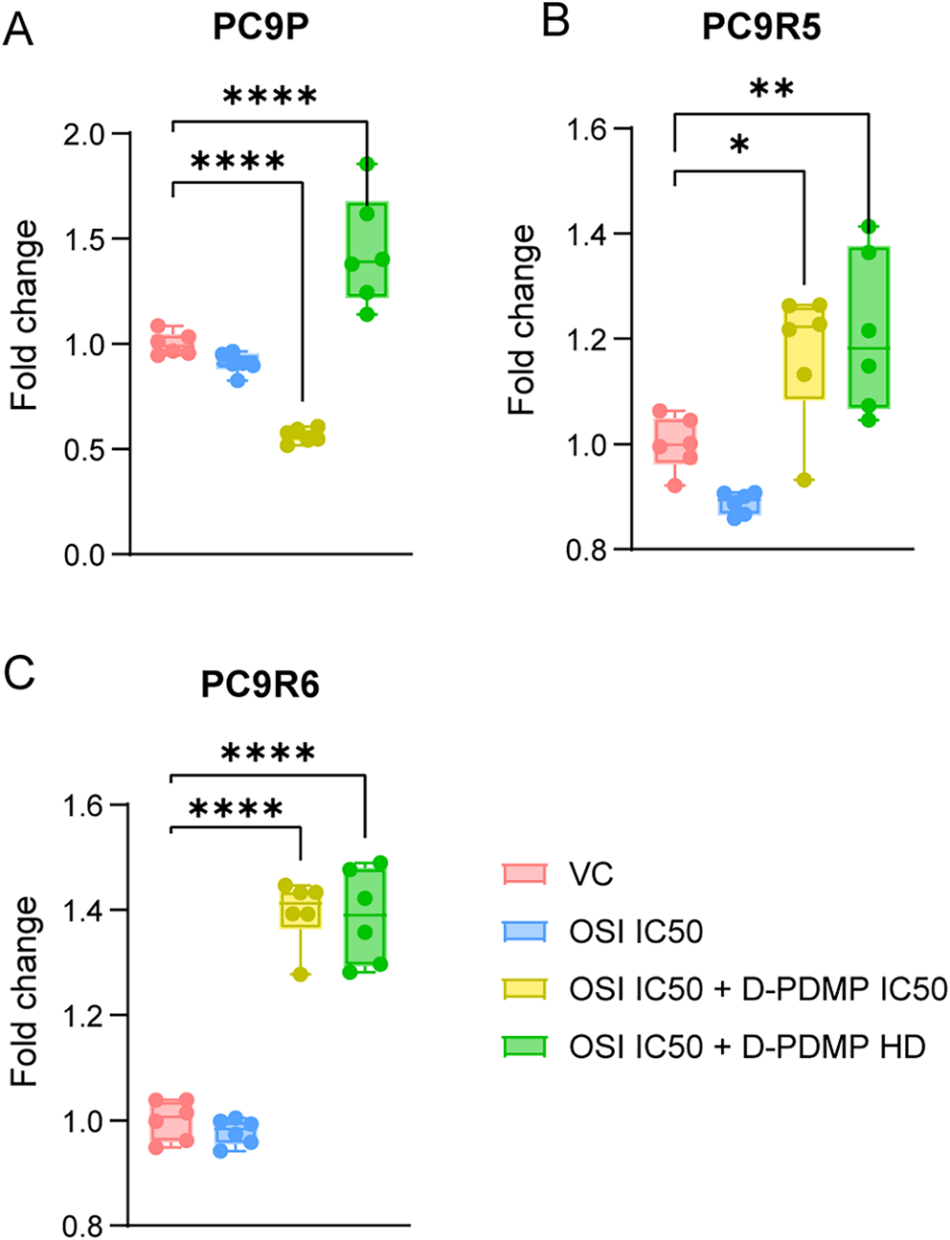
D-PDMP treatment increases free ceramide. Ceramide and glycosphingolipid abundance were measured by LC-MS. Fold change in the ratio of ceramide to total glycosphingolipid relative to vehicle control (VC) in (A) PC9P, (B) PC9R5, and (C) PC9R6 cells. Statistical differences in box plots were determined by two-way ANOVA with Dunnett’s correction of multiple comparisons. ns, *p* > 0.05; *p < 0.05; **p < 0.01; ***p = 0.001; ****p < 0.0001 compared with the control. Data expressed as mean ± SEM. Technical replicates, *n* = 6; data show one representative biological replicate experiment. See Table S1 for dose details.

To confirm our D-PDMP inhibitor experimental results, we additionally increased ceramide levels directly. We treated the OR and OS cell lines with C2-ceramide, a short-chain synthetic analogue of naturally occurring ceramide. We used an SRB assay to determine the maximum nontoxic dose of C2-ceramide and cell-specific IC_50_ values (Figure S4). For each cell line, we treated it with a range of osimertinib doses (0.001–10 μM) and three doses of C2-ceramide: the cell line-specific IC_50_ dose, one lower dose (LD), and one higher dose (HD). Similar to our findings with D-PDMP, the co-treatment of osimertinib with C2-ceramide had a further significant reduction in cell growth, over a range of osimertinib doses, and across all 3 cell lines (Figure S4). However, in PCP9, there was no additional effect on cell growth with C2-ceramide co-treatment above 0.01 μM osimertinib. Notably, both PC9R5 and PC9R6 had a similar decrease in cell growth with C2-ceramide/osimertinib co-treatment, compared to D-PDMP/osimertinib, where a greater reduction in cell growth was achieved in PC9R5.

### Treatment with D-PDMP Increases the Caspase-3/7 Activity in OR and OS Cells

Next, we used the caspase-Glo 3/7 assay to determine if D-PDMP treatment (either as a monotherapy or in combination with osimertinib) increased caspase activity. Each cell line showed caspase activation differing from that of the treatments, with PC9P having the highest caspase activation and PC9R6 having the lowest caspase activation. In PC9P (Figure 6A), the combined treatment of D-PDMP with osimertinib significantly increased caspase activity compared to either drug alone. This was the case for both the D-PDMP IC_50_ and HD treatment groups. In PC9R5, caspase activity was significantly increased only for the D-PDMP (HD) plus osimertinib combination, compared to either monotherapy (Figure 6B). Finally, in PC9R6, caspase activity was significantly increased in both the IC_50_ and HD D-PDMP combination groups when compared to osimertinib monotherapy (Figure 6C). As expected, the PC9P cell line has the highest overall caspase activity, which suggests that D-PDMP treatment increased apoptosis more in the OS cell line. PC9R6 is the OR cell line that is most resistant to osimertinib and has the lowest overall caspase activity, suggesting that it is least susceptible to apoptosis.

**Figure 6:**
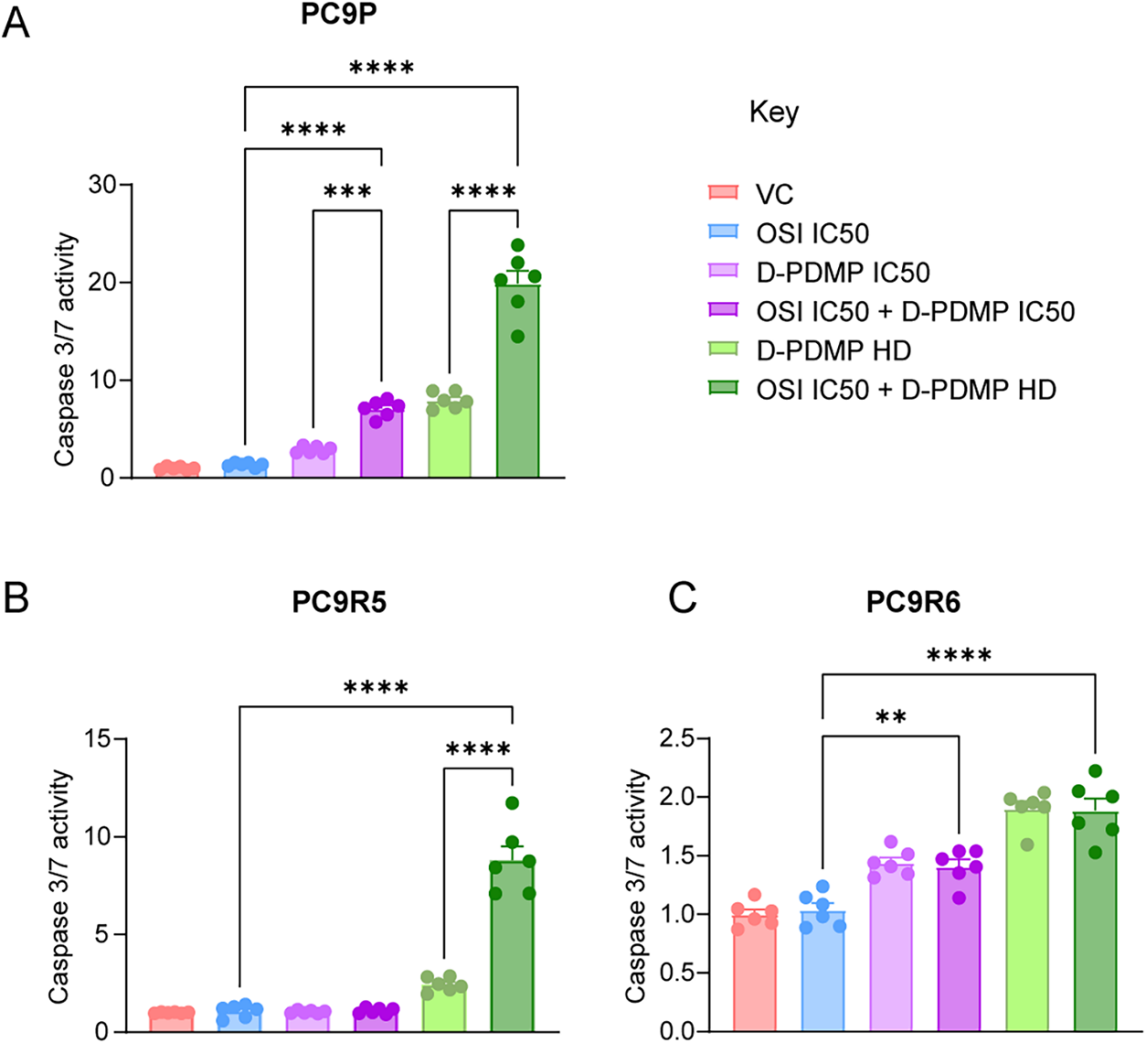
Combination treatment increases caspase-3/7 activity in OR and OS cells. Cell lines (A) PC9P, (B) PC9R5, and (C) PC9R6 were treated with either DMSO (vehicle control; VC), osimertinib IC_50_ monotherapy, D-PDMP IC_50_ monotherapy, osimertinib IC_50_ and D-PDMP IC_50_ combination, D-PDMP HD monotherapy, or osimertinib IC_50_ and D-PDMP HD combination. Caspase activity was measured using the caspase-3/7 Glo assay. Osimertinib IC_50_ was determined for each cell line separately and was 0.003 μM, 1.72 μM, and 1.92 μM for PC9P, PC9R5, and PC9R6 cell lines, respectively. D-PDMP IC_50_/HD were 35/50 μM, 25/50 μM, and 15/30 μM for PC9P, PC9R5, and PC9R6 cell lines, respectively; see also Table S1. Statistical differences in box plots were determined by one-way ANOVA with Dunnett’s correction of multiple comparisons. ns, *p* > 0.05; *p < 0.05; **p < 0.01; ***p = 0.001; ****p < 0.0001. Data expressed as mean ± SEM. Technical replicates, *n* = 6; data is representative of one from 3 biological replicate experiments.

In summary, when compared to the effects of osimertinib monotherapy, the combination of osimertinib and UGCG inhibitor D-PDMP reduced cell growth and increased apoptosis, evidenced by an increase in caspase activity.

### D-PDMP and Osimertinib Combination Act Synergistically in OR Cells

To determine if the effects on cell growth observed with the combination of D-PDMP and osimertinib were synergistic or additive, we treated the OS and OR cell lines with different doses of osimertinib and D-PDMP and calculated the Bliss synergy score. We defined scores of <−5 as antagonistic, −5 to 5 as additive, and >5 as synergistic.^30,44^

In all cell lines, there was a reduction in cell growth with increasing doses of D-PDMP and osimertinib, both individually and in combination (Figure 7). In PC9P, there was no significant synergy between osimertinib and D-PDMP across different combination doses (Figure 7A). This is likely due to the inherent sensitivity of this cell line to osimertinib. In contrast, for the OR cell lines, there was a synergistic effect across several dose combinations (Figure 7B,C). For PC9R5, this was significant in the drug combination of 25 μM D-PDMP and 5 μM osimertinib, compared to osimertinib and D-PDMP monotherapies (Figure 7B). In PC9R6, the Bliss synergy score shows that the combination of D-PDMP and osimertinib acts synergistically, and this increased in a dose-dependent manner (Figure 7C). The combination therapy was significant only compared to D-PDMP monotherapy at higher doses of D-PDMP (Figure 7C).

**Figure 7:**
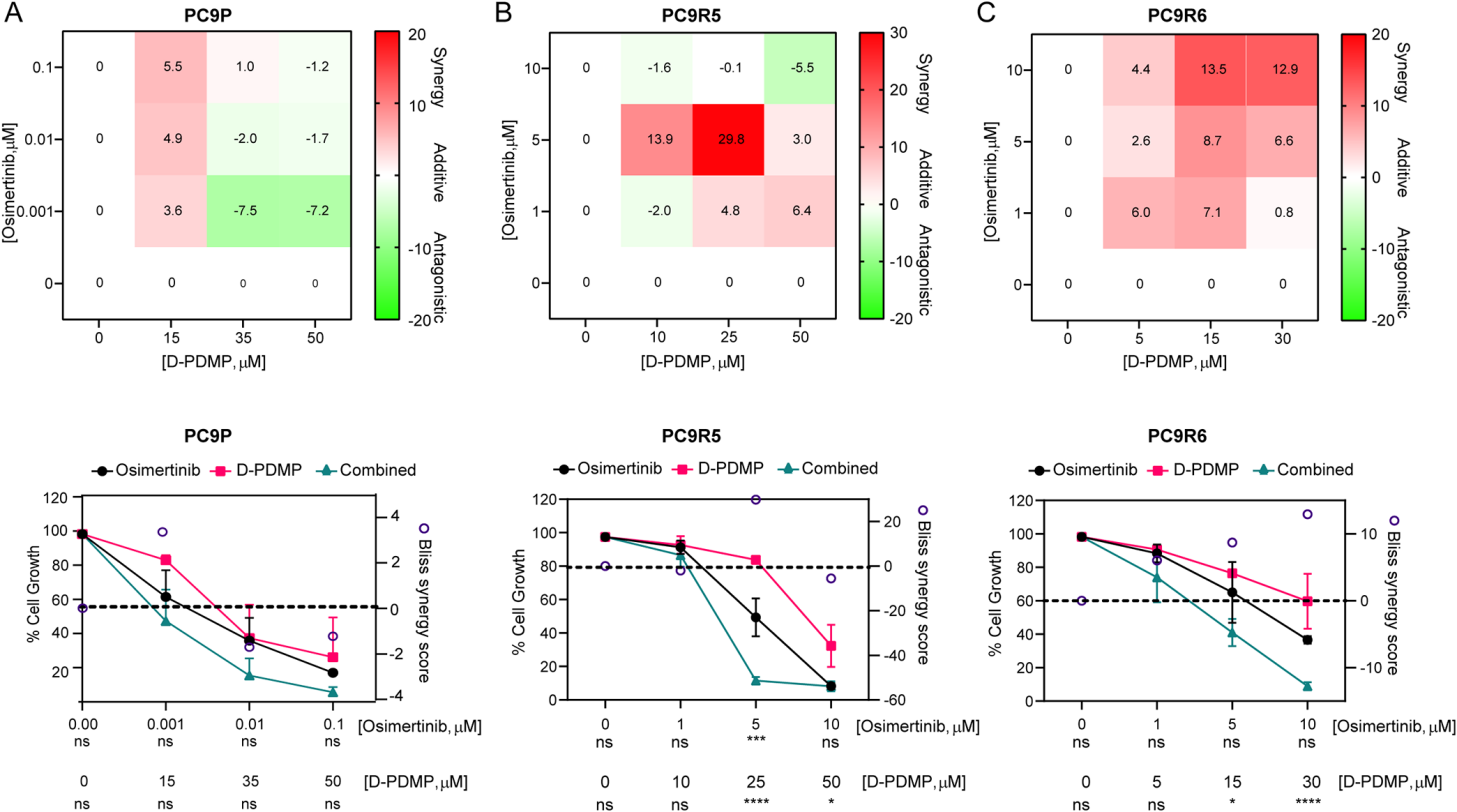
Synergy study of D-PDMP in combination with osimertinib. PC9P (A), PC9R5 (B), and PC9R6 (C) cells were treated with different combinations of D-PDMP and osimertinib for 72 h, after which, cell growth was measured using the SRB assay. Synergy data using the Bliss scoring system at different dose combinations of D-PDMP and osimertinib (upper panel). Effect on cell growth for monotherapies and combinations of D-PDMP and osimertinib (left *y*-axis) with the Bliss synergy score (right *y*-axis) per dose combination (lower panel). Statistical differences determined by two-way ANOVA with Dunnett’s correction of multiple comparisons. ns, *p* > 0.05; *p < 0.05; **p < 0.01; ***p = 0.001; ****p < 0.0001. Data expressed as mean ± SEM. Technical replicates per dose combination, *n* = 3; biological replicates, *n* = 3.

Overall, we have shown that by combining UGCG inhibitor D-PDMP and osimertinib, we can obtain a synergistic effect in terms of cell growth reduction in two OR cell lines.

## Discussion

Here, we have explored the link between dysregulated lipid metabolism and osimertinib resistance, and, importantly, whether we can reinstate osimertinib sensitivity by targeting metabolism. Using pharmacologically induced cell models of OR, we discovered alterations to sphingolipid metabolism at both lipid and protein levels. Studies have shown that dysregulated sphingolipid metabolism is common in cancer, and there is potential for these pathways to be therapeutic targets.^45,46^ Based on our findings, we used the UGCG inhibitor D-PDMP to inhibit the synthesis of glycosphingolipids and increase ceramide levels in OR cells. We hypothesized that the accumulation of ceramide would trigger apoptosis when an extrinsic damaging agent, such as osimertinib, is introduced, thereby reinstating drug sensitivity. Our results demonstrated partial recovery of osimertinib sensitivity through increased free ceramide and apoptosis, as evidenced by increased caspase-3 activity. Furthermore, we established that D-PDMP acted synergistically with osimertinib.

We explored two pharmacologically induced models of OR: PC9R5 and PC9R6. A limitation of this study is that both cell lines are derived from the same parental cell line. However, an advantage of inducing drug resistance pharmacologically is that this would closely mimic the response that patients exhibit to continued and increased administration of osimertinib. Interestingly, while these two cell lines were both phenotypically distinct from the sensitive control, they were also distinct from each other at lipidomic and proteomic levels. However, note that the response to osimertinib was almost identical for the two cell lines.

Differences between the two OR cell lines used here were also noted in the synergy analysis during the combination treatment of D-PDMP and osimertinib. There was a decrease in cell growth in both cell lines at increased doses of both compounds. These effects were found to be synergistic in various combinations of doses in the OR cells. Synergy was dose-dependent in PC9R6 cells. In PC9R5, at the highest doses of both compounds, the interaction appeared to be antagonistic. We suggest this is due to a 90% reduction in cell growth at high doses of osimertinib monotherapy. Therefore, the combination response is less than anticipated by the synergy model.^44^

We investigated the relative proportion of ceramides and glycosphingolipids in OS and OR cell lines when treated with osimertinib monotherapy or a combination of D-PDMP and osimertinib at varying doses specific to each cell line. In all cell lines, the combination treatment groups showed a significant increase in free ceramides. Unexpectedly, in both PC9P and PC9R5 cells, D-PDMP co-treatment resulted in increased GlcCer, the initial product of UGCG. However, LacCer, downstream to GlcCer, was significantly decreased in both OR cell lines with D-PDMP/osimertinib. These findings and a previous study suggest that levels of specific downstream glycosphingolipid species may differ according to the specific OR cell line;^6^ however, overall, the pattern of decreased ceramide and increased total glycosphingolipid is consistent. Increasing doses of D-PDMP have been shown previously in both OR and OS cells to inhibit cell proliferation and colony formation.^8^ UGCG has also been targeted to increase apoptosis in other lung cancer cell lines, such as in A549 cells (human lung adenocarcinoma).^11^ Here, we have shown the effect of inhibiting UGCG on apoptosis in the OR cell lines, revealing a significant increase in caspase activation in all cell lines. In addition to inhibiting UCGC to increase ceramide levels, we also separately treated all cell lines with C2-ceramide, a nonphysiological ceramide analogue, to mimic the effects of intracellular ceramide-mediated death and independently confirm the findings on cell growth with D-PDMP/osimertinib co-treatment.

Beyond the changes in sphingolipid metabolism, we also identified altered levels of plasmalogens (decreased in PCPR5 only), which were supported by changes in protein abundance (decreased in the FAR1/2 in both cell lines). Plasmalogens have been shown to sensitize cells to ferroptosis,^43^ a type of regulated cell death dependent on iron and lipid peroxidation products. Decreased plasmalogens may therefore contribute to survival and drug resistance through reductions in oxidative stress and ferroptosis. PUFAs are susceptible to peroxidation, and their sequestration in TAG (PC9R6 only) could also limit membrane lipid peroxidation. Glutathione is a key metabolic regulator of oxidative stress and an inhibitor of ferroptosis. We found that glutathione metabolism was one of the most enriched pathways in the upregulated proteins common to both OR cell lines compared to their respective OS controls. At the individual protein level, there were changes in several proteins involved in glutathione metabolism. These included increased GPX3 and GPX8 abundance in both OR cell lines compared to the OS control. Both OR cell lines also had increased glutamate-cystine ligases (GCLC and GCLM), which catalyze the rate-limiting step in glutathione synthesis, and increased glutathione reductase (GSR) compared to the control. These proteins play a key role in protecting cells from oxidative stress. In addition, in both OR cell lines, there was increased ferritin (FTH1, FTL) and ferrochelatase (FECH) enzymes that regulate free iron levels in the cell and protect against ferroptosis. Further studies are warranted to provide functional evidence to explore whether ferroptosis is linked to osimertinib resistance.

Our study has several limitations: while the associations between sphingolipid metabolism and osimertinib resistance are strong, it remains to be proven whether these changes are driving resistance or are a consequence of upstream effects. Furthermore, while sphingolipid metabolism and *UGCG* gene expression were shown to be dysregulated across multiple cell types, our inhibitor study was based on only one parental cell line. Lastly, further studies at clinically relevant concentrations for both drugs would be needed in the future to translate our findings. D-PDMP is not currently approved for therapeutic use; however, drug repurposing of other pharmacological inhibitors of UGCG, which are in clinical use (notably miglustat for Gaucher’s disease),^47^ could be a focus of future preclinical studies, along with genetic validation by *UCGC* gene perturbation.

## Conclusion

Our findings indicate that the inhibition of UGCG in pharmacologically induced models of osimertinib resistance resensitizes cells to osimertinib in a combination treatment. These effects are synergistic; we suggest that the mechanism of action is due to increased ceramide levels upon treatment, leading to caspase-3 activation and increased apoptosis. These findings are in line with other studies and indicate a potential novel combination treatment for patients carrying mutations that render them resistant to osimertinib. Through combination therapies that target identified resistance mechanisms such as glycosphingolipid synthesis, we can potentially reinstate the therapeutic effects of osimertinib and prolong treatment success.

## Supplementary Material





## Data Availability

The mass spectrometry proteomics data have been deposited to the ProteomeXchange Consortium via the PRIDE partner repository (http://www.ebi.ac.uk/pride/archive/) with the data set identifier PXD071008. The mass spectrometry lipidomics data have been deposited at the MassIVE repository (https://massive.ucsd.edu/) with the data set identifier MSV000099821.

## Notes

Supporting Information and methods; Figure S1: Dose–response curves for osimertinib treatment; Figure S2: Dose–response curves for D-PDMP treatment; Figure S3: Relative levels of ceramide, GlcCer, and LacCer in osimertinib/D-PDMP-treated cells; Figure S4: C2-ceramide treatment in combination with osimertinib; Table S1: IC_50_ values and doses for combination experiments; Table S2: Gradient elution for lipidomics analysis; Supplementary references (PDF)
Table S3: Full list and identification criteria of all proteins; Table S4: Significantly altered proteins in PC9R5 compared to control; Table S5: Significantly altered proteins in PC9R6 compared to control; Table S6: Lipid-related proteins (XLSX)

## References

[1] Zhou J., Xu Y., Liu J., Feng L., Yu J., Chen D. (2024). Global burden
of lung cancer in 2022 and projections to 2050: Incidence and mortality
estimates from GLOBOCAN. Cancer Epidemiol..

[2] Ganti A. K., Klein A. B., Cotarla I., Seal B., Chou E. (2021). Update of
Incidence, Prevalence, Survival, and Initial Treatment in Patients
With Non–Small Cell Lung Cancer in the US. JAMA Oncol..

[3] Suda K., Mitsudomi T. (2021). Drug Tolerance
to EGFR Tyrosine Kinase Inhibitors in
Lung Cancers with EGFR Mutations. Cells..

[4] Fu K., Xie F., Wang F., Fu L. (2022). Therapeutic strategies for EGFR-mutated
non-small cell lung cancer patients with osimertinib resistance. J. Hematol. Oncol..

[5] Ma Q., Wang J., Ren Y., Meng F., Zeng L. (2020). Pathological
Mechanistic Studies of Osimertinib Resistance in Non-Small-Cell Lung
Cancer Cells Using an Integrative Metabolomics-Proteomics Analysis. J. Oncol..

[6] Martin M. J., Eberlein C., Taylor M., Ashton S., Robinson D., Cross D. (2016). Inhibition of oxidative
phosphorylation suppresses the development
of osimertinib resistance in a preclinical model of EGFR-driven lung
adenocarcinoma. Oncotarget.

[7] Xu C., Zhang L., Wang D., Jiang S., Cao D., Zhao Z., Huang M., Jin J. (2021). Lipidomics reveals
that sustained SREBP-1-dependent lipogenesis is a key mediator of
gefitinib-acquired resistance in EGFR-mutant lung cancer. Cell Death Discovery.

[8] La
Monica S., Vacondio F., Eltayeb K., Lodola A., Volta F., Viglioli M., Ferlenghi F., Galvani F., Galetti M., Bonelli M., Fumarola C., Cavazzoni A., Flammini L., Verzè M., Minari R., Petronini P. G., Tiseo M., Mor M., Alfieri R. (2024). Targeting glucosylceramide synthase induces antiproliferative
and proapoptotic effects in osimertinib-resistant NSCLC cell models. Sci. Rep..

[9] Lin M., Li Y., Wang S., Cao B., Li C., Li G. (2022). Sphingolipid
Metabolism and Signaling in Lung Cancer: A Potential Therapeutic Target. J. Oncol..

[10] Li R. Z., Wang X. R., Wang J., Xie C., Wang X. X., Pan H. D., Meng W. Y., Liang T. L., Li J. X., Yan P. Y., Qu Q. B., Liu L., Yao X. J., Leung E. L. H. (2022). The key role of sphingolipid metabolism
in cancer:
New therapeutic targets, diagnostic and prognostic values, and anti-tumor
immunotherapy resistance. Front. Oncol..

[11] Ravid T., Tsaba A., Gee P., Rasooly R., Medina E. A., Goldkorn T. (2003). Ceramide accumulation precedes caspase-3
activation
during apoptosis of A549 human lung adenocarcinoma cells. Am. J. Physiol. Lung Cell. Mol. Physiol..

[12] Dadsena S., Bockelmann S., Mina J. G. M., Hassan D. G., Korneev S., Razzera G., Jahn H., Niekamp P., Müller D., Schneider M., Tafesse F. G., Marrink S. J., Melo M. N., Holthuis J. C. M. (2019). Ceramides bind VDAC2 to trigger mitochondrial apoptosis. Nat. Commun..

[13] Schömel N., Gruber L., Alexopoulos S. J., Trautmann S., Olzomer E. M., Byrne F. L., Hoehn K. L., Gurke R., Thomas D., Ferreirós N., Geisslinger G., Wegner M. S. (2020). UGCG overexpression leads to increased glycolysis and
increased oxidative phosphorylation of breast cancer cells. Sci. Rep..

[14] Wegner M. S., Gruber L., Mattjus P., Geisslinger G., Grösch S. (2018). The UDP-glucose ceramide glycosyltransferase
(UGCG)
and the link to multidrug resistance protein 1 (MDR1). BMC Cancer..

[15] Eberlein C. A., Stetson D., Markovets A. A., Al-Kadhimi K. J., Lai Z., Fisher P. R., Meador C. B., Spitzler P., Ichihara E., Ross S. J., Ahdesmaki M. J., Ahmed A., Ratcliffe L. E., Christey O’Brien E. L., Barnes C. H., Brown H., Smith P. D., Dry J. R., Beran G., Thress K. S., Dougherty B., Pao W., Cross D. A. E. (2015). Acquired Resistance
to the Mutant-Selective EGFR Inhibitor AZD9291 Is Associated with
Increased Dependence on RAS Signaling in Preclinical Models. Cancer Res..

[16] Shen S., An B., Wang X., Hilchey S. P., Li J., Cao J., Tian Y., Hu C., Jin L., Ng A., Tu C., Qu M., Zand M. S., Qu J. (2018). Surfactant Cocktail-Aided
Extraction/Precipitation/On-Pellet Digestion Strategy Enables Efficient
and Reproducible Sample Preparation for Large-Scale Quantitative Proteomics. Anal. Chem..

[17] Smith P. K., Krohn R. I., Hermanson G. T., Mallia A. K., Gartner F. H., Provenzano M. D., Fujimoto E. K., Goeke N. M., Olson B. J., Klenk D. C. (1985). Measurement
of protein using bicinchoninic acid. Anal. Biochem..

[18] Batth T. S., Tollenaere M. X., Rüther P., Gonzalez-Franquesa A., Prabhakar B. S., Bekker-Jensen S., Deshmukh A. S., Olsen J. V. (2019). Protein
Aggregation Capture on Microparticles Enables Multipurpose Proteomics
Sample Preparation. Mol. Cell Proteomics.

[19] https://www.uniprot.org/proteomes/UP000005640 (accessed October 24, 2024).

[20] Frankenfield A. M., Ni J., Ahmed M., Hao L. (2022). Protein Contaminants Matter: Building
Universal Protein Contaminant Libraries for DDA and DIA Proteomics. J. Proteome Res..

[21] Cox J., Hein M. Y., Luber C. A., Paron I., Nagaraj N., Mann M. (2014). Accurate Proteome-wide
Label-free Quantification by Delayed Normalization
and Maximal Peptide Ratio Extraction, Termed MaxLFQ. Mol. Cell. Proteomics.

[22] Bateman A., Martin M. J., Orchard S., Magrane M., Ahmad S., Alpi E., Bowler-Barnett E. H., Britto R., Bye-A-Jee H., Cukura A., Denny P., Dogan T., Ebenezer T., Fan J., Garmiri P., da Costa Gonzales L.
J., Hatton-Ellis E., Hussein A., Ignatchenko A., Insana G., Ishtiaq R., Joshi V., Jyothi D., Kandasaamy S., Lock A., Luciani A., Lugaric M., Luo J., Lussi Y., MacDougall A., Madeira F., Mahmoudy M., Mishra A., Moulang K., Nightingale A., Pundir S., Qi G., Raj S., Raposo P., Rice D. L., Saidi R., Santos R., Speretta E., Stephenson J., Totoo P., Turner E., Tyagi N., Vasudev P., Warner K., Watkins X., Zaru R., Zellner H., Bridge A. J., Aimo L., Argoud-Puy G., Auchincloss A. H., Axelsen K. B., Bansal P., Baratin D., Batista Neto T. M., Blatter M. C., Bolleman J. T., Boutet E., Breuza L., Gil B. C., Casals-Casas C., Echioukh K. C., Coudert E., Cuche B., de Castro E., Estreicher A., Famiglietti M. L., Feuermann M., Gasteiger E., Gaudet P., Gehant S., Gerritsen V., Gos A., Gruaz N., Hulo C., Hyka-Nouspikel N., Jungo F., Kerhornou A., Le Mercier P., Lieberherr D., Masson P., Morgat A., Muthukrishnan V., Paesano S., Pedruzzi I., Pilbout S., Pourcel L., Poux S., Pozzato M., Pruess M., Redaschi N., Rivoire C., Sigrist C. J. A., Sonesson K., Sundaram S., Wu C. H., Arighi C. N., Arminski L., Chen C., Chen Y., Huang H., Laiho K., McGarvey P., Natale D. A., Ross K., Vinayaka C. R., Wang Q., Wang Y., Zhang J. (2023). UniProt: the Universal Protein Knowledgebase
in 2023. Nucleic Acids Res..

[23] Tyanova S., Temu T., Sinitcyn P., Carlson A., Hein M. Y., Geiger T., Mann M., Cox J. (2016). The Perseus computational
platform for comprehensive analysis of (prote)­omics data. Nat. Methods..

[24] Huang D. W., Sherman B. T., Lempicki R. A. (2009). Systematic
and integrative analysis
of large gene lists using DAVID bioinformatics resources. Nat. Protoc..

[25] Sherman B. T., Hao M., Qiu J., Jiao X., Baseler M. W., Lane H. C., Imamichi T., Chang W. (2022). DAVID: a web server for functional
enrichment analysis and functional annotation of gene lists (2021
update). Nucleic Acids Res..

[26] de
Miguel F. J., Gentile C., Feng W. W., Silva S. J., Sankar A., Exposito F., Cai W. L., Melnick M. A., Robles-Oteiza C., Hinkley M. M., Tsia J. A., Hartley A.-V., Wei J., Wurtz A., Li F., Toki M. I., Rimm D. L., Homer R., Wilen C. B., Xiao A. Z., Qi J., Yan Q., Nguyen D. X., Janne P. A., Kadoch C., Politi K. A. (2023). Mammalian
SWI/SNF chromatin remodeling complexes promote tyrosine kinase inhibitor
resistance in EGFR-mutant lung cancer. Cancer
Cell.

[27] Chambers M. C., Maclean B., Burke R., Amodei D., Ruderman D. L., Neumann S., Gatto L., Fischer B., Pratt B., Egertson J., Hoff K., Kessner D., Tasman N., Shulman N., Frewen B., Baker T. A., Brusniak M. Y., Paulse C., Creasy D., Flashner L., Kani K., Moulding C., Seymour S. L., Nuwaysir L. M., Lefebvre B., Kuhlmann F., Roark J., Rainer P., Detlev S., Hemenway T., Huhmer A., Langridge J., Connolly B., Chadick T., Holly K., Eckels J., Deutsch E. W., Moritz R. L., Katz J. E., Agus D. B., MacCoss M., Tabb D. L., Mallick P. (2012). A cross-platform
toolkit
for mass spectrometry and proteomics. Nat. Biotechnol..

[28] Conroy M. J., Andrews R. M., Andrews S., Cockayne L., Dennis E. A., Fahy E., Gaud C., Griffiths W. J., Jukes G., Kolchin M., Mendivelso K., Lopez-Clavijo A. F., Ready C., Subramaniam S., O’Donnell V. B. (2024). LIPID MAPS: update to databases and tools for the lipidomics
community. Nucleic Acids Res..

[29] Pang Z., Lu Y., Zhou G., Hui F., Xu L., Viau C., Spigelman A. F., MacDonald P. E., Wishart D., Li S., Xia J. (2024). MetaboAnalyst
6.0: towards a unified platform for metabolomics data
processing, analysis and interpretation. Nucleic
Acids Res..

[30] Zheng S., Wang W., Aldahdooh J., Malyutina A., Shadbahr T., Tanoli Z., Pessia A., Tang J. (2022). SynergyFinder
Plus: Toward Better Interpretation and Annotation of Drug Combination
Screening Datasets. Genomics, Proteomics Bioinf..

[31] Malyutina A., Majumder M. M., Wang W., Pessia A., Heckman C. A., Tang J. (2019). Drug combination sensitivity scoring
facilitates the discovery of
synergistic and efficacious drug combinations in cancer. PLoS Comput. Biol..

[32] Rebollido-Rios R., Venton G., Sánchez-Redondo S., Iglesias
I Felip C., Fournet G., González E., Fernández W. R., Escuela D. O. B., Di Stefano B., Panarroche-Díaz R., Martin G., Ceylan I., Costello R., Perez-Alea M. (2020). Dual disruption of aldehyde dehydrogenases
1 and 3 promotes functional changes in the glutathione redox system
and enhances chemosensitivity in nonsmall cell lung cancer. Oncogene.

[33] Song H., Wang Y., Shi C., Lu J., Yuan T., Wang X. (2021). SH3KBP1 Promotes Glioblastoma Tumorigenesis
by Activating EGFR Signaling. Front. Oncol..

[34] Go G. W., Mani A. (2012). Low-density lipoprotein
receptor (LDLR) family orchestrates cholesterol
homeostasis. Yale J. Biol. Med..

[35] Luo X., Li N., Zhao X., Liao C., Ye R., Cheng C., Xu Z., Quan J., Liu J., Cao Y. (2019). DHRS2 mediates cell
growth inhibition induced by Trichothecin in nasopharyngeal carcinoma. J. Exp. Clin. Cancer Res..

[36] Lee J. S., Lee H., Lee S., Kang J. H., Lee S. H., Kim S. G., Cho E. S., Kim N. H., Yook J. I., Kim S. Y. (2019). Loss of
SLC25A11 causes suppression of NSCLC and melanoma tumor formation. EBioMedicine.

[37] Mascaux C., Iannino N., Martin B., Paesmans M., Berghmans T., Dusart M., Haller A., Lothaire P., Meert A. P., Noel S., Lafitte J. J., Sculier J. P. (2005). The role of RAS
oncogene in survival of patients with lung cancer: A systematic review
of the literature with meta-analysis. Br J.
Cancer.

[38] Huang Z. C., Li H., Sun Z. Q., Zheng J., Zhao R. K., Chen J., Sun S. G., Qu C. J. (2018). Distinct prognostic roles of HSPB1
expression in non-small cell lung cancer. Neoplasma.

[39] Sun N., Zhao X. (2022). Argininosuccinate synthase 1, arginine deprivation
therapy and cancer
management. Front. Pharmacol..

[40] Liu J. Z., Hu Y. L., Feng Y., Jiang Y., Guo Y. B., Liu Y. F., Chen X., Yang J. L., Chen Y. Y., Mao Q. S., Xue W. J. (2020). BDH2 triggers
ROS-induced cell death
and autophagy by promoting Nrf2 ubiquitination in gastric cancer. J. Exp. Clin. Cancer Res..

[41] An B. C., Choi Y. D., Oh I. J., Kim J. H., Park J. I., Lee S. (2018). GPx3-mediated redox
signaling arrests the cell cycle and acts as
a tumor suppressor in lung cancer cell lines. PLoS One.

[42] Honsho M., Fujiki Y. (2023). Regulation of plasmalogen
biosynthesis in mammalian
cells and tissues. Brain Res. Bull..

[43] Zou Y., Henry W. S., Ricq E. L., Graham E. T., Phadnis V. V., Maretich P., Paradkar S., Boehnke N., Deik A. A., Reinhardt F., Eaton J. K., Ferguson B., Wang Q., Fairman J., Keys H. R., Dančík V., Clish C. B., Clemons P. A., Hammond P. T., Boyer L. A., Weinberg R. A., Schreiber S. L. (2020). Plasticity of ether lipids promotes
ferroptosis susceptibility and evasion. Nature.

[44] Yadav B., Wennerberg K., Aittokallio T., Tang J. (2015). Searching for Drug
Synergy in Complex Dose–Response Landscapes Using an Interaction
Potency Model. Comput. Struct. Biotechnol. J..

[45] Janneh A. H., Ogretmen B. (2022). Targeting Sphingolipid
Metabolism as a Therapeutic
Strategy in Cancer Treatment. Cancers.

[46] Companioni O., Mir C., Garcia-Mayea Y., Leonart M. E. (2021). Targeting Sphingolipids for Cancer
Therapy. Front. Oncol..

[47] Barth B. M., Shanmugavelandy S. S., Tacelosky D. M., Kester M., Morad S. A. F., Cabot M. C. (2013). Gaucher’s
Disease and Cancer: A Sphingolipid
Perspective. Crit. Rev. Oncog..

