# Supplementary material for: Altered Sphingolipid Metabolism is Associated with Osimertinib Resistance in Nonsmall-Cell Lung Cancer
Source: J Proteome Res. 2026 Apr 16;25(5):2569–80. doi: 10.1021/acs.jproteome.6c00216 (PMC13140601; doi:10.1021/acs.jproteome.6c00216)
Supplement: Supplementary file 1 [file pr6c00216_si_001.pdf]

Altered sphingolipid metabolism is associated with osimertinib resistance in non-small cell lung cancer

Julia Babuta[1], Aleksandra Gruevska[1], Chiharu Wickremesinghe[2], Alex Montoya[3], Georgia Roumelioti[3], Pavel Shliaha[3], Cristina Balcells[2], Flora McKinney[2], Chandler Bray [1], Toby Athersuch[1], Matthew Martin[4], Hector Keun[2]*, Zoe Hall[1,]*

[1] *Department of Metabolism, Digestion and Reproduction, Imperial College London, London, United Kingdom*

[2] *Department of Surgery and Cancer, Imperial College London, London, United Kingdom*

[3] *MRC Laboratory of Medical Sciences, London, United Kingdom*

[4] *AstraZeneca, Biomedical Campus, Cambridge, United Kingdom*

Supporting Information

- **Supplementary material and methods**
- **Figure S1:** Dose response curves for osimertinib treatment in PC9P, PC9R5 and PC9R6 cells.
- **Figure S2:** Dose response curves for D-PDMP treatment in PC9P, PC9R5 and PC9R6 cells.
- **Figure S3:** Relative levels of ceramide, GlcCer and LacCer in cells treated with osimertinib monotherapy or osimertinib and D-PDMP.
- **Figure S4:** C2-ceramide treatment in combination with osimertinib.
- **Table S1:** Cell-line specific IC50 values for osimertinib and D-PDMP doses for combination experiments.
- **Table S2:** Mobile phase gradient program for lipidomics analysis
- **Tables S3-S6** are in "Supporting information file 2.xls" (**Table S3**: Full list and identification criteria of all proteins**; Table S4**: Significantly altered proteins in PC9R5 compared to control; **Table S5**: Significantly altered proteins in PC9R6 compared to control; **Table S6**: Lipid-related proteins)
- **Supplementary references**

Supplementary Materials and Methods

Sulphorodamine B (SRB) cell growth assay for cytotoxicity and dose response

SRB assay was used to determine cell viability and inhibition of cell growth. Cells (PC9P, PC9R5, PC9R6) were seeded in 96-well plates at a density of 20,000 cells/well in 200μL of respective media. Cell counts were performed using the Vi-Cell XR Cell Viability Analyzer (Beckman Coulter). Cells lines were incubated at 37 °C with 5 % CO_2 for 24 hours. Spent medium was removed and cells were then incubated in 100uL of media containing osimertinb (0.0001, 0.001, 0.01, 0.1, 1, and 10 μM) or D-PDMP (10 μM, 20 μM, 30 μM, 40 μM and 50 μM) and vehicle control (0.1 % DMSO) for 72 hours. Six technical replicates were used for each condition.

Cells were fixed by addition of 10 % (w/v) trichloroacetic acid (TCA; 25 μL) solution (Sigma-Aldrich, T6399) to each well and left to incubate at 4 °C for 1 hour. The plate was washed using cold water to remove the TCA and incubated at room temperature overnight. Cells were then stained with 100 μL of 0.4 % (w/v) SRB solution (Sigma-Aldrich, 230162) in 1 % (v/v) acetic acid (ThermoFisher, 10005920) for 30 minutes before being aspirated off the cells and the plate was rinsed with 1% acetic acid (v/v) to remove all unbound dye. Plates were incubated at room temperature overnight. 200 μL of 10 mM Tris Base solution (Sigma-Aldrich, T1699) was added to each well and left on a shaker for 30 minutes to mobilise the bound SRB dye. A second plate identical to the first was set up with 200 μL of 10mM Tris Base solution, and 50 μL of the original plate was added to this second plate and pipetted up and down to dilute the sample 1:5. Absorbance was measured using a CLARIOstar plate reader (BMG LABTECH), with OD set to 545 nm. Cell free wells were used as blanks to remove background noise. Values were normalised to vehicle control and plotted using GraphPad Prism v10.2.0. Experiments were repeated in triplicate.

Combination treatment of D-PDMP with osimertinib

Cell lines (PC9P, PC9R5 and PC9R6) were seeded in 96-well plates at a density of 20,000 cells/well in 200 µL of respective media. Cells lines were incubated at 37 °C with 5 % CO_2 for 24 hours. PC9P cells were treated with vehicle, osimertinib (0.001µM - 10 µM) and/or D-PDMP (15 µM, 35 µM and 50 µM) for 72 hours. PC9R5 cells were treated with vehicle, osimertinib (0.001µM - 10 µM) and/or D-PDMP (10 µM, 25 µM and 50 µM) for 72 hours. PC9R6 cells were treated with vehicle, osimertinib (0.001µM - 10 µM) and/or D-PDMP (5 µM, 15 µM and 30 µM) for 72 hours. Six technical replicates were used per condition. Data was acquired using the SRB assay described above. Experiments were repeated in triplicate.

Caspase-Glo® 3/7 Assay

PC9P, PC9R5 and PC9R6 cells were cultured in 96 well plates, one plate was used for an SRB assay and another white bottomed plate was used for the Caspase-Glo® 3/7 Assay (Promega, G8092). 24 hours after seeding, cells were treated with either vehicle, osimertinib and/or D-PDMP for 72 hours. After treatment, the SRB plates were fixed and processed using the described SRB assay protocol. The plate for the Caspase-Glo® 3/7 Assay were processed following manufacturers guidelines. Luminescence was measured using a CLARIOstar Plate Reader (BMG Labtech). Caspase activity was background corrected using cell-free wells and normalised to the SRB cell mass data and to VC wells. Six technical replicates were used per condition. Experiments were repeated in triplicate.

Proteomics analysis via LC-MS

Protein digests were analysed by LC-MS using an UltiMate 3000 RSLC nano liquid chromatography system coupled to an Exploris 240 MS (Thermo Scientific) via an EASY-Spray source. Electrospray nebulisation was achieved by interfacing to Bruker PepSep emitters (PN: PSFSELF20, 20 µm). Peptides were separated using a 66-minute stepped gradient method in positive ion mode only using an Acquity CSH C18 1.7 µM beads, 300

µM x 35cm column, where 2 µg of digests resuspended in 5 µL were injected directly into the column at a flow rate of 5 µL/min for 4 minutes. Mobile phases were prepared with UPLC grade solvents and chemicals. Mobile phase A was 95% H_2O + 5 % DMSO + 0.1% formic acid (v/v). Mobile phase B was 75 % ACN + 20 % H_2O + 5 % DMSO + 0.1 % formic acid (v/v). The peptides were separated using a 66-minute stepped gradient of 0-45 % Mobile Phase B. The data-independent acquisition (DIA) mode was as follows: an initial MS1 scan with a mass range of 410-1650 m/z at 120,000 resolution with an AGC target of 3x106 ions for a maximum injection time (IT) of 200 ms. This was followed by an MSX (boxcar) MS1 scan with 10 variable windows covering a range of 410-1650 m/z at 120,000 resolution and 310 DIA scans with variable window width at 30,000 resolution. The ACG target was again set to $3x10^6$ ions, with maximum IT set to auto. The normalised collision energy was set to 27 % and the total run acquisition time was 82 minutes.

Sample preparation for lipidomics

Cells were cultured in 6cm Cornell plates (6 technical replicates) for 48 hours. Media was removed and the cells were washed with 1 mL cold PBS. 1 mL of ice-cooled HPLC grade methanol was added and the cells were scraped and collected. A further 500 µL methanol was added, scraped and collected. The samples were then dried under nitrogen. Folch extraction was used to then obtain the organic layer. 100 µL of a pre-mixed internal standard and 950 µL of 2:1 chloroform/methanol was added to the dried down samples. Samples were sonicated for 5 minutes. 400 µL HPLC grade water was added to each sample and then vortexed for 20 seconds, followed by centrifugation at 13,000 rcf for 10 minutes. The bottom organic layer and the protein pellet was dried down under nitrogen and stored at -80 °C. On the day of analysis, the organic layer was reconstituted in 100 µL of 2:1 chloroform/methanol and diluted 1:10 in 2:1:1 isopropanol /acetonitrile/water.

The internal standard mix (ISM) was prepared as follows: stock solutions of 10mg/mL each were diluted in methanol to form the final ISM. The mix included N-palmitoyl-

d31-D-erythro-sphingosine (16:0-d31 Ceramide), pentadecanoic-d29 acid (15:0-d29 FFA), heptadecanoic-d33 acid (17:0-d33 FFA), eicosanoic-d39 acid (20:0-d39 FFA), 1-palmitoyl(D31)-2-oleyl-sn-glycero-3-phosphatidylcholine (16:0-d31-18:1 PC), 1-palmitoyl(d31)-2-oleyl-sn-glycero-3-phosphoethanolamine (16:0-d31-18:1 PE), 1-palmitoyl-d31-2-oleoyl-sn-glycero-3-[phospho-rac-(1-glycerol)] (16:0-d31-18:1 PG), N-palmitoyl(d31)-d-erythro-sphingosylphosphorylcholine (16:0-d31 SM), lysophosphatidylcholine (LPC) 14:0-d29 LPC-d13, glyceryl tri(pentadecanoate-d29) (45:0-d87 TAG), and glyceryl-tri(hexadecanoate-d31) (48:0-d93 TAG).

Lipid analysis via untargeted reverse phase LC-MS

Lipid extracts were analysed by liquid chromatography-mass spectrometry (LC-MS) using an UltiMate 3000 HPLC system coupled to an LTQ-Orbitrap Elite MS system (Thermo Scientific). A 10-minute reverse phase liquid chromatographic method was used to separate lipid species prior to detection by MS in both positive and negative ionisation modes. Separation was achieved using an Acquity UPLC C18 BEH column, 130Å, 1.7 µm, 2.1 mm X 50 mm, 1/pk (Waters Corporation). Mobile phases were prepared with UPLC grade solvents and chemicals. Mobile phase A/C was 60 % ACN, 40 % H20 with 10 mM ammonium formate (positive ion mode) or 10 mM ammonium acetate (negative ion mode). Mobile phase B/D was 90 % IPA, 10 % ACN with 10 mM ammonium formate (positive ion mode) or 10 mM ammonium acetate (negative ion mode). The mobile phase gradient elution for positive and negative 10-minute reverse phase mode can be found in **Table S2**. 5 µL of each sample was injected in positive mode and 10 µL was injected in negative mode, both at a flow rate of 0.5 mL/min at a column temperature of 55 °C. QC pooled samples were used in all experiments and injected every 5 samples to measure quality throughout the run. CID MS/MS fragmentation was performed on the QC sample only. A blank which was taken through all sample preparation steps was injected every 5 samples and used for data clean-up. The spectra were acquired in both ionization modes in the range of 110-2000 m/z at 60,000 mass resolution.

Processing and analysis of lipidomics data

Data was acquired using Xcalibur (Thermo) and converted from a raw format to mzML via ProteoWizard MS Convert software[1]. The mzML files were processed using XCMS package in R studio (Version R4.2.2.2), using R scripts developed in-house. Features were retained if the average signal was more than 3 times the signal of the blank and less than 20 % RSD for 5 QC repeat injections. Data were normalised to the total ion count (TIC). Lipids were annotated based on retention time patterns and accurate mass database searches in Lipid Maps® "Bulk" Structure Searches Database (LMSD)[2]. Metaboanalyst v6.0 was used for hierarchical clustering with Euclidean algorithm for distance measure, Ward algorithm for clustering measure and ANOVA to test signficance. GraphPad Prism v10.2.0 was used to generate any box plots.

Synergy analysis

Synergy data analysis was performed on different drug combination data of both D-PDMP and osimertinib, obtained via SRB assay, to determine if the combinations were synergistic or simply additive. The web application SynergyFinder+[3] was used with the Bliss Independence model of combination analysis chosen. This plaform uses statistical synergy models to score each combination. There are four main models used; highest single agent (HSA), Loewe additivity (Loewe), Bliss independence (Bliss) and zero interaction (ZIP)[3]. We selected the Bliss synergy score model as this model is more appropriate when the drugs work in different pathways and work independently of each other[4]. The cut-offs for Bliss scoring we used were as follows: < -5 is antagonistic, -5 to 5 is additive and > 5 is synergistic[5].

The principle of Bliss scoring is shown below[5]:

$$S_{Bliss} = y_c - (y_1(x_1) + y_2(x_2) - y_1(x_1)y_2(x_2))$$

Where;

- x_1 = Drug A at a specific concentration,
- x_2 = Drug B at a specific concentration,
- y_c = combined effect of x_1 and x_2,
- $y_1(x_1)$ = single drug effect of Drug A,
- $y_2(x_2)$ = single drug effect of Drug B.

Supplementary Figures and Tables

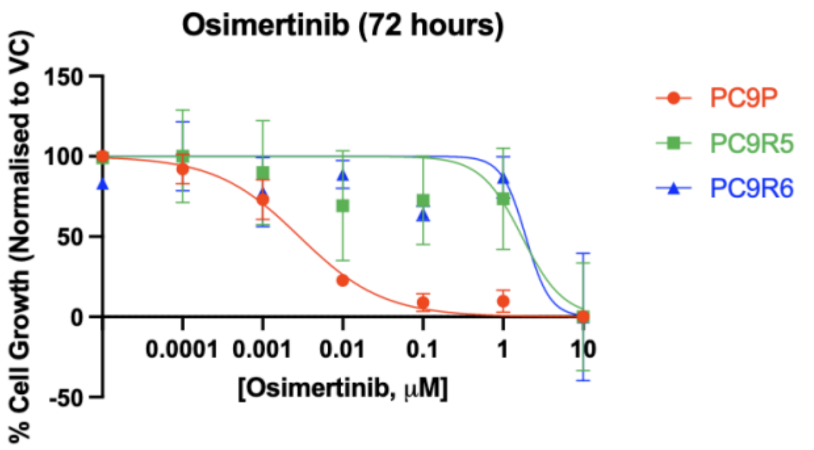

Figure S1. Dose response curves for osimertinib treatment in PC9P, PC9R5 and PC9R6 cell lines. Cells were treated with 0-10 µM osimertinib for 72 hours before cell growth was measured using SRB assay. Data expressed as mean ± SEM (technical replicates, n=6; biological replicates, n = 3).

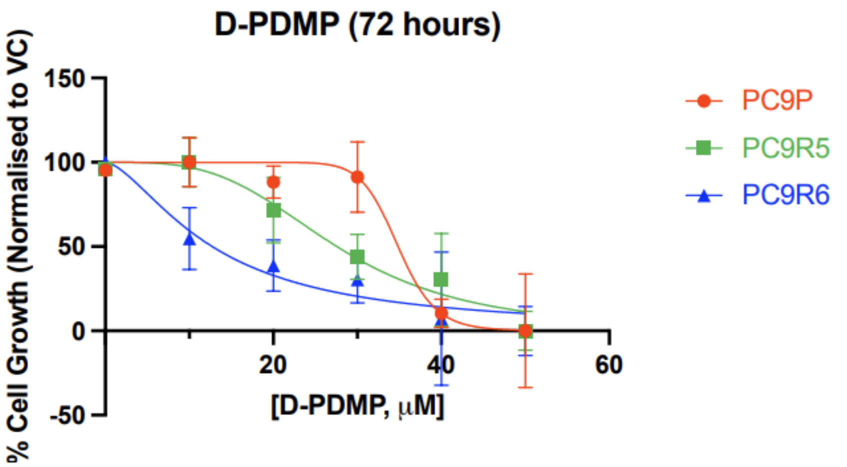

Figure S2. Dose response curves for D-PDMP treatment in PC9P, PC9R5 and PC9R6 cells. Cell lines were treated with D-PDMP for 72 hours before cell growth was measured using SRB assay. Data expressed as mean ± SEM (technical replicates, n=6; biological replicates, n = 3).

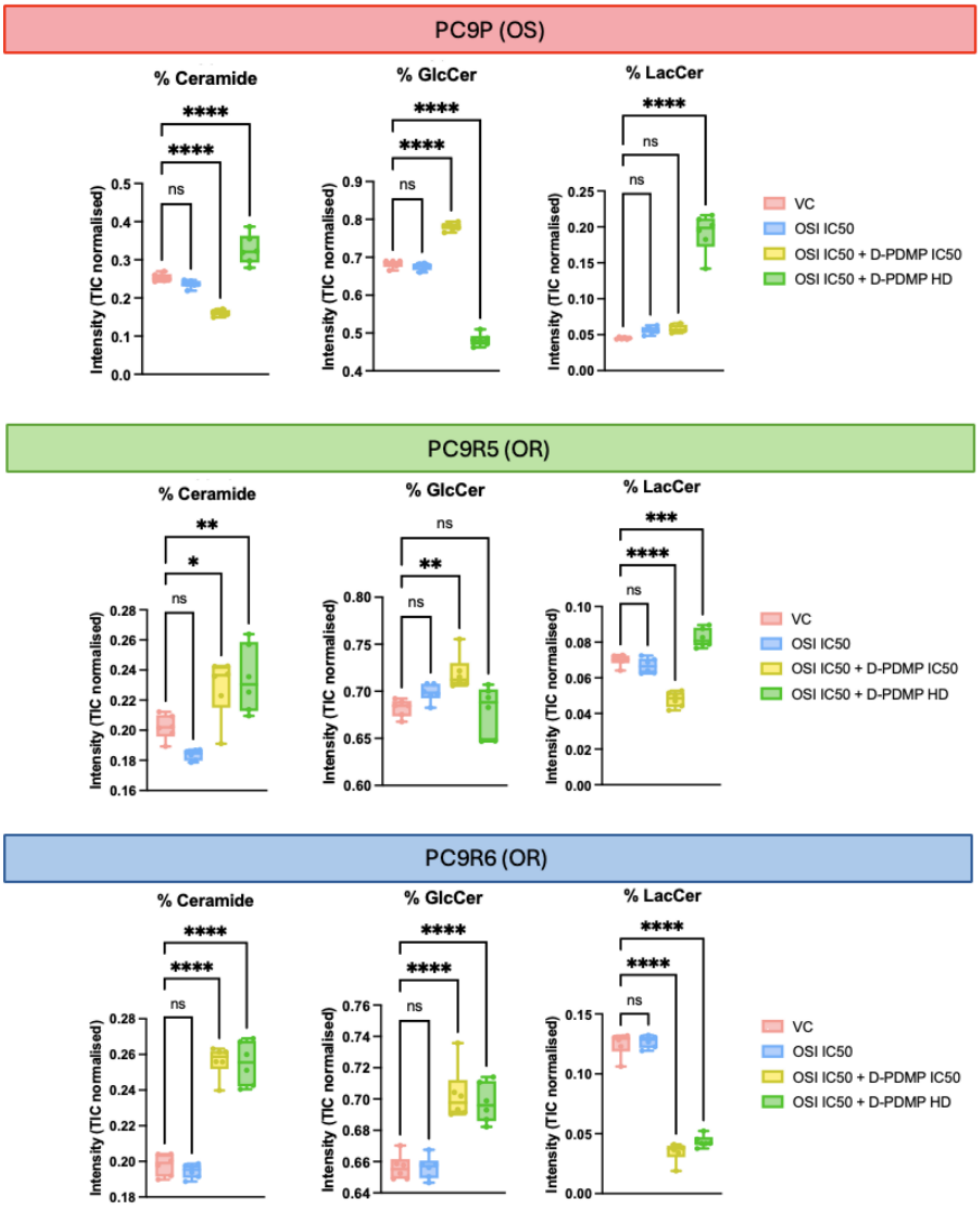

Figure S3. Relative levels of ceramide, GlcCer and LacCer in cells when treated with DMSO vehicle control (VC), osimertinib monotherapy and combination doses of osimertinib/D-PDMP (see also **Table S1)**. Data was normalised to total ion count (TIC). Statistical differences determined by one-way ANOVA with Dunnet's correction for multiple comparisons. ns $p > 0.05$, * $p < 0.05$, ** $p < 0.01$, *** $p = 0.001$, **** $p < 0.0001$ compared with control. Data expressed as mean ± SEM (technical replicates, n = 6; data shows one representative biological replicate experiment from n=3).

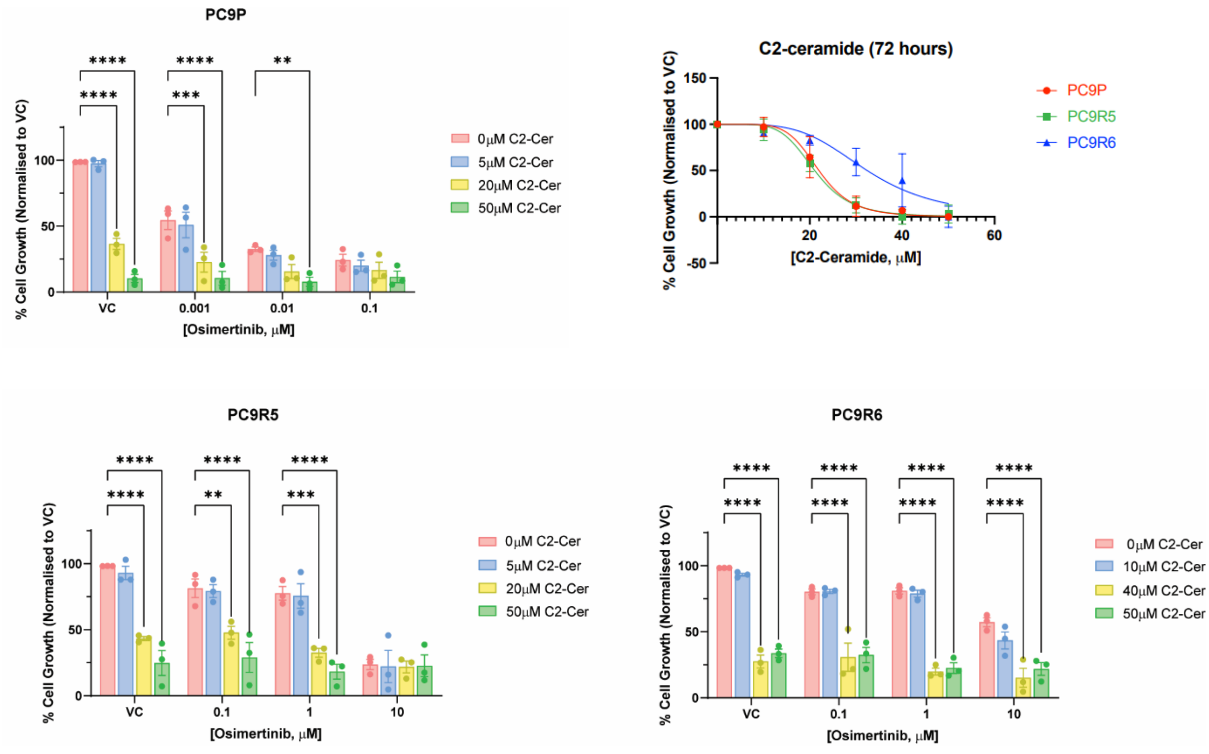

Figure S4. C2-ceramide treatment in combination with osimertinib. Percentage (%) change in growth, measured via an SRB assay, after cells were treated with various combinations of C2-ceramide and osimertinib for 72 hours. PC9P, PC9R5 and PC9R6 cells were treated with DMSO vehicle control (VC), osimertinib monotherapy, C2-ceramide monotherapy and combinations of both drugs for 72 hours. C2-ceramide LD/IC_{50}/HD were 5/20/50 μM, 5/20/50 μM and 10/40/50 μM for PC9P, PC9R5 and PC9R6 cell lines, respectively. Statistical differences in box plots determined by two-way ANOVA with Dunnet's correction for multiple comparisons. ns $p > 0.05$, * $p < 0.05$, ** $p < 0.01$, *** $p = 0.001$, **** $p < 0.0001$ compared with VC/osimertinib monotherapy. Data expressed as mean ± SEM (technical replicates, n = 6, biological replicates, n = 3).

Table S1. Cell-line specific IC_{50} values for osimertinib and D-PDMP doses for combination experiments - IC_{50}, lower dose (LD) and higher dose (HD).

Cell line	Osimertinib IC_{50} (μM)	D-PDMP IC_{50} (μM)	D-PDMP LD (μM)	D-PDMP HD (μM)
PCP9 (OS)	0.003	35	15	50
PCPR5 (OR)	1.72	25	10	50
PCPR6 (OR)	1.92	15	5	30

Table S2. Mobile phase gradient elution for lipids in positive (A/B) and negative ionisation (C/D) modes.

Time (mins)	Flow rate (mL/min)	Mobile phase A/C (%)	Mobile phase B/D (%)
0	0.5	60	40
0.8		57	43
0.9		50	50
4.8		46	54
4.9		30	70
5.8		19	81
8		1	99
8.5		1	99
8.6		60	40
10		60	40

Supplementary References

1 Chambers MC, Maclean B, Burke R, Amodei D, Ruderman DL, Neumann S, Gatto L, Fischer B, Pratt B, Egertson J, Hoff K, Kessner D, Tasman N, Schulman N, Frewen B, Baker TA, Brusniak MY, Paulse C, Creasy D, Flashner L, Kani K, Moulding C, Seymour SL, Nuwaysir LM, Lefebvre B, Kuhlmann F, Roark J, Rainer P, Detlev S, Hemenway T, Huhmer A, Langridge J, Connolly B, Chadick T, Holly K, Eckels J, Deutsch EW, Moritz RL, Katz JE, Agus DB, MacCoss M, Tabb DL, Mallick P. A cross-platform toolkit for mass spectrometry and proteomics. Nat Biotechnol. 2012, 30, 918.

2 Conroy MJ, Andrews RM, Andrews S, Cockayne L, Dennis EA, Fahy E, Gaud C, Griffiths WJ, Jukes G, Kolchin M, Mendivelso K, Lopez-Clavijo AF, Ready C, Subramaniam S, O'Donnell VB. LIPID MAPS: update to databases and tools for the lipidomics community. Nucleic Acids Res. 2024, 52, D1677.

3 Zheng S, Wang W, Aldahdooh J, Malyutina A, Shadbahr T, Tanoli Z, Pessia A, Tang J. SynergyFinder Plus: Toward Better Interpretation and Annotation of Drug Combination Screening Datasets. Genomics, Proteomics & Bioinformatics. 2022, 20, 587.

4 Liu Q, Yin X, Languino LR, Altieri DC. Evaluation of Drug Combination Effect Using a Bliss Independence Dose–Response Surface Model. Statistics Biopharm Res. 2018, 10, 112.

5 Malyutina A, Majumder MM, Wang W, Pessia A, Heckman CA, Tang J. Drug combination sensitivity scoring facilitates the discovery of synergistic and efficacious drug combinations in cancer. PLoS Comput Biol. 2019, 15, e1006752.
